# Comparative efficacy of onsite, digital, and other settings for cognitive behavioral therapy for insomnia: a systematic review and network meta-analysis

**DOI:** 10.1038/s41598-023-28853-0

**Published:** 2023-02-02

**Authors:** Laura Simon, Lisa Steinmetz, Bernd Feige, Fee Benz, Kai Spiegelhalder, Harald Baumeister

**Affiliations:** 1 Department of Clinical Psychology and Psychotherapy, Institute of Psychology and Education, University Ulm, Lise-Meitner-Str. 16, 89081 Ulm, Germany; 2 Department of Psychiatry and Psychotherapy, Medical Center–University of Freiburg, Faculty of Medicine, University of Freiburg, Freiburg, Germany

**Keywords:** Public health, Medical research, Psychiatric disorders

## Abstract

Given the limited availability and accessibility of onsite cognitive behavioral therapy for insomnia (CBT-I), other CBT-I settings, such as internet-delivered CBT-I (iCBT-I), have been proposed. The primary aim of the study was to compare the efficacy of available CBT-I settings on insomnia severity. A systematic review and frequentist network meta-analysis of available CBT-I settings was performed. PsycINFO, PsycARTICLES, MEDLINE, PubMed, and CINAHL were searched for randomized controlled trials (RCTs) investigating any CBT-I settings in adults with insomnia disorder. The systematic literature search (3851 references) resulted in 52 RCTs. For the primary outcome insomnia severity, all examined CBT-I settings yielded significant effects when compared to WL. Large standardized mean differences were found for telehealth (− 1.35;95%CI − 1.73, − 0.97), individual onsite CBT-I (− 1.30;95%CI − 1.51, − 1.09), guided bibliotherapy (− 1.05;95%CI − 1.38, − 0.71), smartphone (− 1.04;95%CI − 1.62, − 0.46), group-delivered CBT-I (− 1.01;95%CI − 1.21, − 0.82), and unguided iCBT-I (− 1.01;95%CI − 1.20, − 0.82). Guided iCBT-I (− 0.73;95%CI − 0.95, − 0.51) and unguided bibliotherapy (− 0.67;95%CI − 1.00, − 0.35) yielded medium effect sizes. The results underline that health care systems should intensify their efforts to provide synchronously-delivered CBT-I (individual onsite, group-delivered, and telehealth), and particularly individual onsite CBT-I, given its solid evidence base. Medium to large effect sizes for iCBT-I and guided bibliotherapy indicate that self-help settings may be a viable alternative when synchronously-delivered CBT-I is not available.

## Introduction

Insomnia disorder is one of the most prevalent mental disorders^1^. It is estimated that up to a third of primary care patients fulfill the diagnostic criteria^2,3^. The disorder has a high burden of disease and impacts the quality of life and daytime functioning^4^. Moreover, insomnia is a risk factor for other somatic and mental health conditions^5–7^ and creates high societal costs due to work absenteeism and presenteeism as well as health care usage^8,9^. Given its high prevalence and impact on an individual and societal level, the treatment of insomnia disorder is of high public health relevance.

Yet, the provision of care for insomnia disorder, which is currently mainly conducted by primary care physicians, is a major challenge for health care systems. Clinical guidelines recommend cognitive behavioral therapy for insomnia (CBT-I) as the first-line treatment^10,11^. However, given the scarce availability and accessibility of trained CBT-I providers, only a fraction of the people suffering from insomnia disorder receive guideline-compliant treatment^12,13^. As a result, alternative CBT-I settings have been proposed to enhance the dissemination of CBT-I. These proposed settings range from self-help programs, such as internet-delivered CBT-I (iCBT-I) or bibliotherapy to settings where patients and therapists communicate via video or chat. Pairwise meta-analyses provide preliminary support for some of these alternative settings^14–17^. However, evidence comparing alternative CBT-I settings to the first-line way of providing CBT-I in an individual onsite setting is scarce.

For depressive disorder and other mental health and somatic conditions, preliminary evidence indicates that digitalized cognitive behavioral therapy (CBT), at least if therapeutically guided, can be as effective as onsite CBT^18–20^. Across all investigated mental health and somatic conditions, the overall results of Carlbring et al. indicated equivalence between digitalized and onsite CBT^20^. Yet, in the case of insomnia, their analysis of two studies pointed to a larger effect of onsite CBT-I. However, this effect did not reach significance. Moreover, subgroup analyses of two recent meta-analyses indicated that onsite CBT-I may be superior to self-help CBT-I^15,21^.

On a study level, studies comparing efficacy between various CBT-I settings are limited, and the existing evidence is contradictory. One study indicated the noninferiority of guided iCBT-I when comparing it to onsite group-delivered CBT-I (group)^22^. Moreover, no significant differences between unguided iCBT-I and individual onsite CBT-I (F2F) were found in a study of military personnel^23^. While these studies point to a comparable efficacy of onsite CBT-I and iCBT-I, two other studies indicate that onsite CBT-I may be superior to iCBT-I. In a comparison of guided iCBT-I, F2F, and waiting list control (WL), F2F yielded larger effects at all assessment points^24^. Moreover, a recent noninferiority trial comparing unguided iCBT-I to F2F found significantly lower levels of insomnia severity for F2F^25^.

A better understanding of the comparative efficacy of available CBT-I settings would contribute to the discussion how alternative CBT-I settings can improve the care for insomnia and how resources should be allocated. Network meta-analyses offer a possibility to compare different interventions/settings, even if they have not been investigated head to head in randomized controlled trials (RCTs)^26^.

Hence, the present study aims to compare the efficacy of CBT-I settings using the framework of a frequentist network meta-analysis. The following specific research questions are addressed by synthesizing RCTs examining adult patients with insomnia disorder:

1. How do CBT-I settings compare in their efficacy on insomnia severity measured via self-report questionnaires?
2. How do CBT-I settings compare in their efficacy on sleep quality as well as on subjectively reported (i.e., via sleep diary or self-report questionnaire), and objectively measured (i.e., via polysomnography or actigraphy) sleep-related outcomes (i.e., total sleep time, sleep efficiency, sleep onset latency, and wake after sleep onset)?
3. How do CBT-I settings compare in their efficacy on response, remission, and intervention completion rates?

Given the contradictory existing evidence, no a-priori hypotheses have been set.

## Methods

### Search strategy and selection criteria

This systematic review and frequentist network meta-analysis was conducted according to the PRISMA extension statement for network meta-analyses (Supplementary Appendix S1)^27^.

The databases PsycINFO, PsycARTICLES, MEDLINE, PubMed, and CINAHL were searched for publications from 1987, which is the publication date of DSM-III-R^28^, until November 23rd, 2021. Terms indicative of insomnia disorder, CBT-I, and CBT-I components were combined for the search string. The search string per database is detailed in Supplementary Appendix S2. The electronic database searches were supplemented with manual searches for published, unpublished, and ongoing RCTs in ClinicalTrials.gov, by screening the reference lists of included studies, and by contacting experts of the field (i.e., the European Insomnia Network).

Only RCTs published in English or German were eligible for inclusion. Participants were adult patients with insomnia disorder diagnosed following the DSM-5^29^, DSM-IV-TR^30^, DSM-IV^31^, DSM-III-R^28^, or consistent criteria. Studies defining comorbid conditions or shift work as inclusion criteria were excluded. Thus, comorbid conditions were allowed, provided they were not an inclusion criterion for the respective study. Interventions of interest were CBT-I, which was conceptualized as interventions incorporating at least one cognitive component (e.g., cognitive restructuring, cognitive control, paradoxical intention, worry time), one behavioral component (i.e., stimulus control, sleep restriction), and education about sleep. CBT-I was not allowed to be investigated in combination with other treatments (e.g., bright light therapy, pharmacotherapy). Eligible comparison conditions were either another CBT-I setting or sleep hygiene education (SHE), psychological placebo, WL, treatment as usual (TAU), or active contact control. Studies were ineligible if they compared, according to our categorization (see Table 1), the same CBT-I settings with varying intensity against each other (e.g., varying intensities of guidance in guided iCBT-I as in^32,33^) without another comparison condition. Outcome measures eligible for inclusion were standardized self-report questionnaires or data from actigraphy/polysomnography for insomnia severity, sleep quality, subjectively reported or objectively measured sleep parameters (i.e., total sleep time, sleep efficiency, sleep onset latency, wake after sleep onset), response, remission, or intervention completion rates. A detailed description of the outcome data is provided in Supplementary Table S1.

**Table 1:** CBT-I settings and control groups.

Settings/control groups (nodes)	Abbreviation	Definition	Number of studies investigating this setting
Individual onsite CBT-I	F2F	CBT-I is provided to an individual patient in a clinical setting by a trained health care provider	16
Group-delivered CBT-I	group	CBT-I is provided to a group of patients in a clinical setting by a trained health care provider	14
Unguided bibliotherapy	booklet	CBT-I is provided by reading materials used by patients in their homes without any additional clinical support from a health care provider	4
Guided bibliotherapy		CBT-I is provided by reading materials used by patients in their homes augmented with clinical support (e.g., via telephone) from a health care provider	4
Unguided internet-delivered CBT-I	unguided iCBT-I	CBT-I is provided via a website/web application on an internet browser without any additional clinical support from a health care provider; animated virtual coaches fell into this category	16
Guided internet-delivered CBT-I	guided iCBT-I	CBT-I is provided via a website/web application on an internet browser augmented with clinical support (e.g., via E-Mails; telephone) from a health care provider	10
Smartphone-delivered CBT-I	smartphone	CBT-I is provided via a smartphone application without any additional clinical support from a health care provider	1
Telehealth-delivered CBT-I	telehealth	CBT-I is provided in real-time by a trained health care provider via video or chat	4
Sleep hygiene education	SHE	Sleep hygiene education may be provided in varying settings (e.g., flyer, E-Mail, browser-based) and in varying intensities and consist of general recommendations on lifestyle and environmental factors that may promote or interfere with sleep	13
Active contact control	ActCon	Compromised control conditions where participants either engaged in a self-monitoring control (e.g., weekly sleep diaries during the intervention period) or in which participants were contacted by the research staff	6
Psychological placebo	placebo	Credible intervention without a known active therapeutic ingredient (e.g., imagery relief therapy)	2
Treatment as usual	TAU	Participants did not receive any additional treatment for their insomnia but were explicitly permitted to obtain additional help from their primary caregiver	2
Waiting list	WL	Participants were granted access to CBT-I after the intervention period	24

Classification of the CBT-I settings was adapted from^34^. The classification of the settings might differ from the original labeling in the study. In^35,36^ patients also had the option to access the intervention using a smartphone. Given that there was no further differentiation between settings in the outcome data, we categorized these interventions as unguided iCBT-I.

Identified records were managed using Citavi^37^. After manually removing duplicates, titles and abstracts of the identified studies were screened. LSi and LSt independently selected the studies. Outcome data (expressed as means and standard deviations) were independently extracted by LSi and LSt. Conflicts were resolved by discussion. If means and standard deviations were not provided, they were calculated from the available statistical indices as described in the Cochrane Handbook^38^. Information on study design features, sample characteristics, and intervention characteristics were extracted.

### Data analysis

The primary outcome was the standardized mean difference (SMD) from pre- to post-treatment of insomnia severity measured via self-report. All standardized self-report questionnaires measuring insomnia severity were allowed. See Supplementary Table S1 for an overview of the secondary outcomes.

Data was prepared for the analysis using Python^39^ and analyzed using R (version 4.2.0^40^). Random-effect frequentist network meta-analyses were fitted using the R package *netmeta* (version 2.1), which automatically accounts for multi-arm studies^41,42^. SMDs with 95% confidence intervals (CI) were calculated for all continuous outcomes. Dichotomous data were transformed using the Freeman-Tukey double arcsine transformation to calculate SMDs. WL was used as the reference treatment in all forest plots. P-Scores were used to estimate the relative rankings within the frequentist network^43^.

CBT-I settings and control conditions were classified according to the description provided in each study. Table 1 details all possible nodes. To visualize the network, network plots were created using the function netgraph() of the R package *netmeta* (version 2.1)^41^.

### Assessment of heterogeneity and inconsistencies

A common estimate for the heterogeneity variance was assumed for all comparisons. The presence of statistical heterogeneity and inconsistencies were assessed using Higgins's I^2^ and Cochran's Q (Q_withindesigns_ for a test of heterogeneity within designs and Q_betweendesigns_ for a test of inconsistencies between designs). Moreover, heterogeneity and inconsistencies were analyzed via independent path decompositions visualized by net heat plots^44^.

### Risk of bias and publication bias

Risk of bias was assessed independently by LSi and LSt using the Cochrane Risk of Bias tool 2 (RoB 2.0^45^) for the primary outcome insomnia severity. Risk of bias was evaluated for (1) the randomization process, (2) deviations from the intended intervention, (3) missing outcome data, (4) measurement of the outcome, and (5) selection of the reported outcome. Any discrepancies were resolved by consensus and arbitration by consulting a third reviewer (FB). We decided against calculating an overall risk of bias rating following the recommendations by Jüni et al.^46^. Comparison-adjusted funnel plots comparing CBT-I settings against the control conditions were produced to explore publication bias or other small-study effects.

The study was prospectively registered on the Open Science Framework (https://osf.io/py4eq). There were no deviations from the study registration. There was no funding source for this study.

## Results

**Figure 1:**
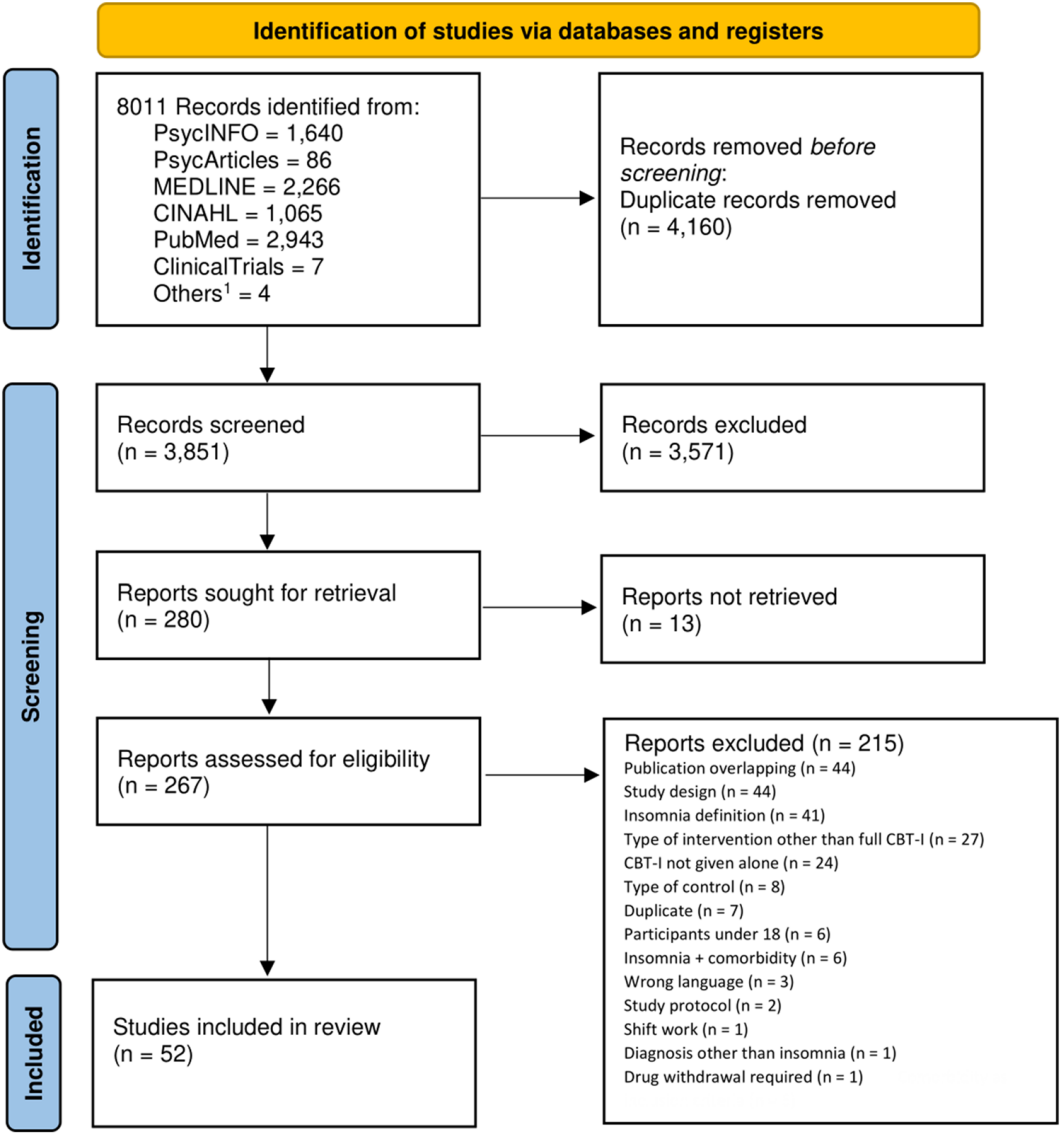
Study selection process. ^1^by contacting experts of the field (i.e., the mailing list of the European Insomnia Network). Adapted from: Page et al.^93^.

The systematic literature search identified a total of 3851 references. Finally, 52 studies^22–25,35,36,47–92^, including 12,544 participants, fulfilled our eligibility criteria and were included in this network meta-analysis. The detailed study selection process and reasons for exclusion are outlined in the PRISMA flow chart in Fig. 1. Characteristics of the included studies are reported in Supplementary Table S2, and details on the interventions and control conditions with their classification into the respective nodes of the network are reported in Table 1. The included studies had a median sample size of 91 participants (range 10 to 3755), with a mean age of 43.4 years and 70.6% being female.

**Figure 2:**
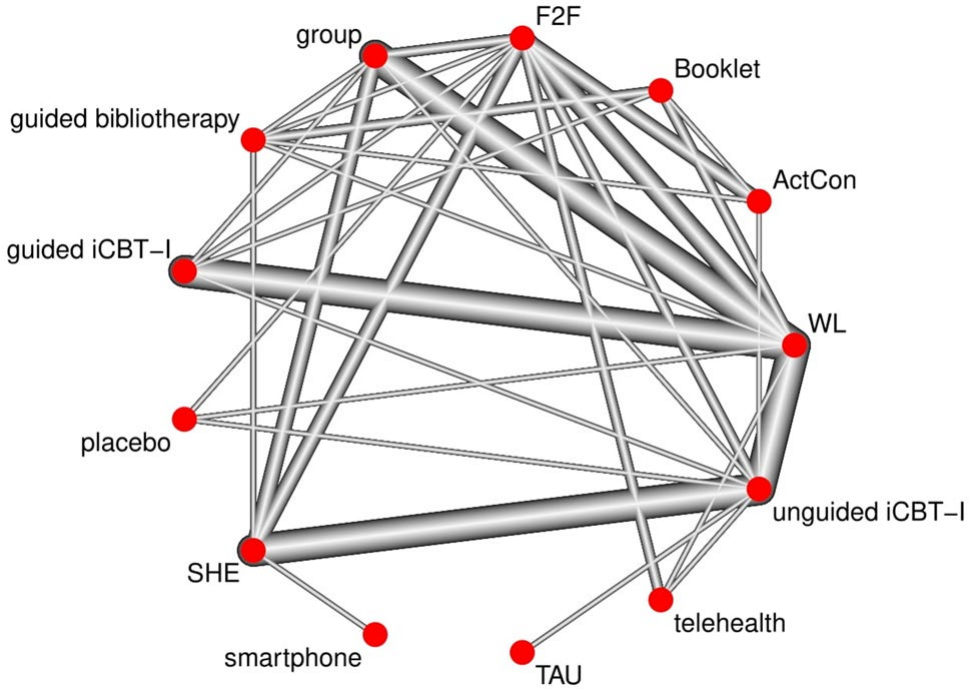
Network plot insomnia severity. The most direct evidence came from the pairwise comparisons of guided iCBT-I to WL, unguided iCBT-I to WL, and group to WL. Smartphone was not strongly attached to the network, with only one study comparing it to SHE. The network plots were created using the function netgraph() of the R package *netmeta* (version 2.1^41^) in the software R (version 4.2.0^40^).

**Figure 3:**
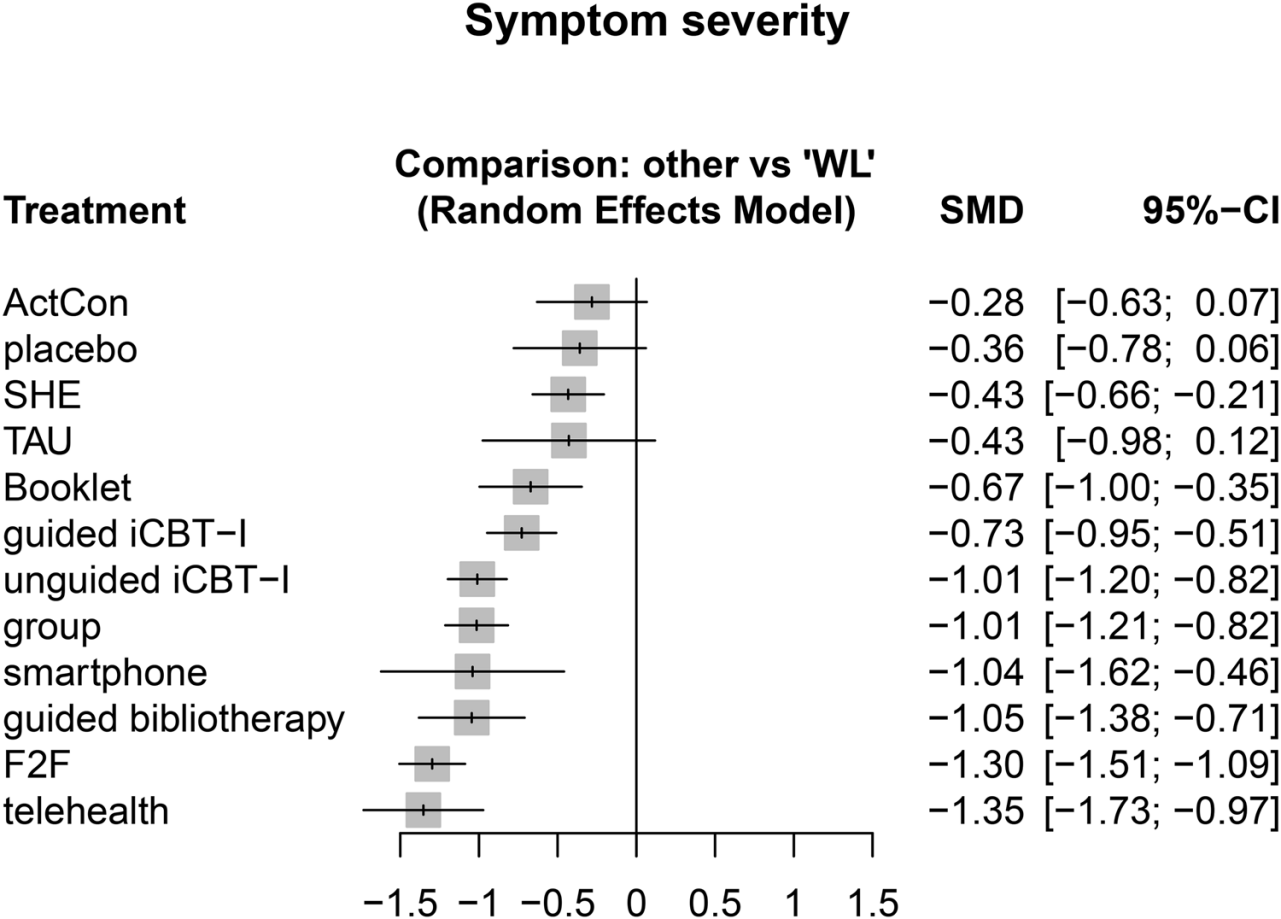
Forest plot insomnia severity. Treatments were ranked according to their P-Score.

Forty-four studies investigating a total of 4662 participants were included in the network meta-analysis for insomnia severity. The following self-report questionnaires were used to measure insomnia severity: the Insomnia Severity Index^94^ (k = 37), the Insomnia Symptom Questionnaire^95^ (k = 3), the Sleep Condition Indicator^96^ (k = 2), the Athens Insomnia Scale^97^ (k = 1), and the eight-item insomnia subscale of the SLEEP-50^98^ (k = 1). A well-connected network (Fig. 2) was found for the primary outcome insomnia severity. The network consisted of 13 nodes and was based on 66 pairwise comparisons. Figure 3 shows the forest plot presenting the SMDs of all available settings compared to WL. Results indicated significant effects of all examined CBT-I settings. Neither of the CBT-I settings was superior to another. Large effect sizes were found for telehealth (− 1.35, 95%CI − 1.73 to − 0.97), F2F (−1.30, 95%CI − 1.51 to − 1.09), guided bibliotherapy (− 1.05, 95%CI − 1.38 to − 0.71), smartphone (− 1.04, 95%CI − 1.62 to − 0.46), group (− 1.01, 95%CI − 1.21 to − 0.82), and unguided iCBT-I (− 1.01, 95%CI − 1.20 to − 0.82). Both guided iCBT-I (− 0.73, 95% CI − 0.95 to − 0.51) and unguided bibliotherapy (− 0.67, 95%CI − 1.00 to − 0.35) yielded medium effect sizes. P-Scores were the largest for telehealth and F2F (0.94 and 0.92, respectively; Supplementary Appendix S3). Substantial heterogeneity and inconsistencies were found (I^2^  = 77.7%; Q_withindesigns_ = 42.12, p = .0006; Q_betweendesigns_  =  150.12, p  <  .0001). Inconsistencies and sources of heterogeneity were explored using net heat plots (Supplementary Fig. S2).

The network graphs of the secondary outcomes, the forest plots presenting the SMDs of all available nodes compared to WL, the respective net heat plots, information on the network meta-analyses, and P-Scores are presented in the supplementary material (Supplementary Figs. S1 and S2, Supplementary Table S3 and Supplementary Appendix S3).

Results indicated significant effects of all examined CBT-I settings except unguided bibliotherapy for subjective sleep quality. Medium effect sizes were found for guided bibliotherapy (0.73, 95%CI 0.40 to 1.06), F2F (0.71, 95%CI 0.49 to 0.92), smartphone (0.68, 95%CI 0.14 to 1.21), and group (0.68, 95%CI 0.42 to 0.93). For the subjective total sleep time, significant effects for unguided bibliotherapy (0.28, 95%CI 0.15 to 0.41), group (0.27, 95%CI 0.14 to 0.39), and guided iCBT-I (0.21, 95%CI 0.12 to 0.30) were found. For subjective sleep efficiency, significant effects were found for all examined CBT-I settings except unguided bibliotherapy. A large effect size was found for group (0.85, 95%CI 0.59 to 1.12), medium effect sizes were found for guided bibliotherapy (0.70, 95%CI 0.30 to 1.09), F2F (0.52, 95%CI 0.25 to 0.78), and telehealth (0.52, 95%CI 0.07 to 0.96). Results indicated significant effects of all examined CBT-I settings on subjective sleep onset latency. Medium effect sizes were found for guided bibliotherapy (− 0.49, 95%CI − 0.69 to − 0.29), telehealth (− 0.45, 95%CI − 0.68 to − 0.22), group (− 0.40, 95%CI: − 0.51 to − 0.29), and F2F (− 0.40, 95%CI − 0.53 to − 0.27). Results indicated significant effects of all examined CBT-I settings on subjective wake after sleep onset. Medium effect sizes were found for group (− 0.64, 95%CI − 0.85 to − 0.44), telehealth (− 0.61, 95%CI − 1.05 to − 0.16), and F2F (− 0.48, 95%CI: − 0.71 to − 0.26). All examined CBT-I settings except telehealth yielded a significant reduction of the objective total sleep time compared to WL. Medium negative effect sizes were found for group (− 0.73, 95%CI − 0.95 to − 0.51), unguided iCBT-I (− 0.61, 95%CI: − 1.08 to − 0.15), and F2F (− 0.51, 95%CI − 0.81 to − 0.22). None of the examined CBT-I settings yielded significant effects on objective sleep efficiency. Significant effects of F2F (− 0.47, 95%CI − 0.75 to − 0.18), telehealth (− 0.55, 95%CI − 0.97 to − 0.14), and SHE (− 0.38, 95%CI − 0.70 to − 0.05) on objective sleep onset latency were found. None of the examined CBT-I settings yielded significant effects on objective wake after sleep onset. Significant effects of guided bibliotherapy (0.89, 95%CI 0.36 to 1.41), telehealth (0.77, 95%CI 0.02 to 1.51), F2F (0.69, 95%CI 0.18 to 1.19), group (0.49, 95%CI 0.17 to 0.82), and guided iCBT-I (0.30, 95%CI 0.04 to 0.56) were found for response rates. Significant effects for remission rates were found for telehealth (0.88, 95%CI 0.15 to 1.60), F2F (0.76, 95%CI 0.27 to 1.25), guided bibliotherapy (0.66, 95%CI 0.24 to 1.09), and unguided iCBT-I (0.39, 95%CI 0.03 to 0.74). Results showed significant negative effects for group (− 0.38, 95%CI − 0.63 to − 0.13), unguided iCBT-I (− 0.29, 95%CI − 0.52 to − 0.06), and guided iCBT-I (− 0.25, 95%CI − 0.49 to − 0.02) on intervention completion rates.

A summary graph of the RoB 2.0 rating and a detailed rating for all included studies per domain is provided in the supplementary material (Supplementary Figs. S3 and S4). The most common sources of risk of bias were missing outcome data and bias in the measurement of the outcome. The comparison-adjusted funnel plots (Supplementary Fig. S5) of all examined outcomes appeared symmetrical.

## Discussion

Given the current treatment gap that can be partially attributed to the low scalability of onsite CBT-I, it is crucial to compare the potential of alternative CBT-I settings. Across outcomes, we did not find evidence for the superiority of any CBT-I setting. For the primary outcome insomnia severity, large effect sizes were found for telehealth, F2F, guided bibliotherapy, smartphone, group-delivered CBT-I, and unguided iCBT-I. Guided iCBT-I and unguided bibliotherapy yielded medium effect sizes. Similarly, across most other subjective sleep-related outcomes, F2F, group, guided bibliotherapy, and telehealth yielded the largest effect sizes and largest P-Scores.

Consistent with a previous meta-analysis on objective sleep-related outcomes^99^, no significant effects were found for objective sleep efficiency and wake after sleep onset. Medium negative effects, thus a reduction of objective total sleep time, were found for all settings except for telehealth. This reduction may be due to sleep restriction and stimulus control which aim to increase sleep pressure^100^. Conversely, small positive effects for subjective total sleep time, indicating increases, were found for unguided bibliotherapy, group, and guided iCBT-I, which may be attributable to a decrease of the subjective–objective sleep discrepancy that has been observed after CBT-I^101^. The settings SHE, telehealth, and F2F yielded a small effect on objective sleep onset latency. In general, the results align with existing evidence indicating that the observed effects of CBT-I are more substantial in self-reported outcome parameters compared to objectively measured outcome parameters^99^.

Guided bibliotherapy yielded a large effect size for response, and telehealth yielded a large effect size for remission. However, given the limited number of studies investigating these outcomes and the limited number of studies investigating guided bibliotherapy and telehealth, these results should be interpreted carefully. Interestingly, group had a larger negative effect size for intervention completion than guided or unguided iCBT-I. However, the network meta-analysis on intervention completion rates should be interpreted with caution, given the heterogeneous reporting of these data.

In accordance with previous meta-analyses, onsite CBT-I (i.e., group and F2F) tended to yield larger effects than iCBT-I^15,21^. As F2F was ranked among the first for the majority of outcomes and given its solid evidence base, the expansion of F2F in all health care systems appears to be crucial. Group may be a resource-saving alternative if few CBT-I providers are available. In this context, intervention retention should be closely monitored, given that attrition rates may potentially be elevated in a group setting. While only four of the included studies investigated telehealth, the results point to the potential of telehealth. As telehealth could be particularly relevant for (remote) areas where the number of qualified CBT-I providers is low, improving the evidence base for this setting seems timely.

Where an expansion of synchronous communication settings (i.e., F2F, group, or telehealth) is not possible or only possible to a limited extent, it should be built upon the potential of self-help settings that have been proven to be effective. This study's findings align with previous pairwise meta-analyses confirming the efficacy of self-help CBT-I^14–16,102^. Guidance might be an important factor in bibliotherapy, as guided bibliotherapy demonstrated a larger effect size and higher P-score compared to unguided bibliotherapy. However, it must be noted that only four studies investigated guided bibliotherapy. Guided bibliotherapy might be an interesting alternative for patients who are skeptical of digitalized CBT-I programs or in areas with limited internet- and end-user device coverage. However, as therapists have no access to patients' progress in the intervention unless patients share it, guidance (e.g., via telephone) might be difficult in routine care. Hence, further investigations on the efficacy and feasibility of guided bibliotherapy are desirable.

Our analyses and current evidence^14–17^ indicate that guided and unguided iCBT-I should be considered to improve the dissemination of CBT-I in routine care. In line with the literature, medium to large effect sizes were found for most subjective sleep-related outcomes for guided and unguided iCBT-I. Interestingly, unguided iCBT-I was comparable to guided iCBT-I for most outcomes and achieved a large effect size for insomnia severity, whereas guided iCBT-I yielded a medium effect size. These findings contrast a recently published network meta-analysis where guided iCBT-I achieved a higher ranking than unguided iCBT-I for subjective sleep-related outcome data^103^. Hasan et al.^103^ classified iCBT-I programs featuring virtual therapists (e.g.,^35,36,53,76,104^) as guided. In line with other works investigating the impact of guidance^105,106^, we defined iCBT-I programs as guided if they featured some kind of human support due to the influence of human support on scalability. Thus, all iCBT-I programs that supported patients solely using automated processes were classified as unguided in our study, even if they provided individualized support to the patients (e.g., via tailoring the intervention/using virtual therapists). By employing this node specification criterion, interventions that vary in their intensity of individualized support were lumped together. While it seems likely that the intensity of individualized support influences the examined outcomes, we decided against splitting the nodes for two reasons: First, information describing the intensity of support is often limited, impacting the feasibility to distinguish reliably between low and high intensities. Second, given that the nodes were already relatively small, a further subdivision could have impacted the network symmetry and the estimations. Nevertheless, given the medium to large effect sizes, iCBT-I, and in particular unguided iCBT-I, could have a major impact on the diminishment of the treatment gap and thus reach patients who would currently not receive any CBT-I.

Yet, for the implementation of iCBT-I in routine care, attrition appears to be a major challenge. Our analysis of the intervention completion rates underlines previous works that patients in iCBT-I are likely to terminate the intervention prematurely^107^. Interestingly, our analysis indicated that guided and unguided iCBT-I achieved comparable effects on intervention completion. Thus, automated individualized support may achieve similar results as the support provided by a human. Nonetheless, one has to keep in mind that we are speaking of comparable low and not comparable high completion rates. Particularly as it can be expected that intervention completion rates will be even lower in real-world settings compared to research settings^108^. This highlights the need to expand investigations of factors influencing attrition and possible countermeasures. A scoping review indicated the potential of various engagement strategies (e.g., personalization, peer support, gamification)^109^. However, the review also illustrated that experimental investigations of the effectiveness of the various engagement strategies are scarce. Therefore, research should focus on factors influencing attrition, how engagement strategies could foster retention in iCBT-I and whether they differ between guided and unguided iCBT-I.

The evidence base of this systematic review and network meta-analyses has some important limitations that should be considered when interpreting the results. First, some CBT-I settings were less commonly investigated than others. For example, only one study examined smartphone^79^, four guided bibliotherapy^48,51,68,80^, and four telehealth^49,61,62,66^. Moreover, for some outcomes (e.g., insomnia severity), the network was based on data from many studies and consisted of many different nodes, while for other outcomes (e.g., objective sleep-related outcomes), few data existed, and consequently, the corresponding networks were rather small. Furthermore, the included studies yielded a considerable risk of bias, particularly because rates of missing outcome data were high. Additionally, substantial heterogeneity and inconsistencies were found in several of the examined outcomes. While we set a strict a-priori definition of full CBT-I, there was still variance in the components comprising CBT-I, which may have contributed to both statistical and clinical heterogeneity. The differing implementation of the CBT-I settings, particularly of iCBT-I, may have contributed to the heterogeneity. In many research articles, the description of the settings (e.g., description of guidance in iCBT-I) was rather scarce, which may have led to the wrong categorization of interventions. Thus, future studies should provide a detailed description of study design features that may affect the outcomes, making also further subdivisions (e.g., on the intensity of individualized support) feasible. The differential efficacy of available CBT-I settings across subgroups (e.g., insomnia patients on or off sleep medication, patients with mental or somatic comorbidity, etc.) is crucial to give insights on which treatment is best for the individual patient. Yet, we decided against conducting further subgroup analyses as subgroup analyses result in split networks and thus data loss and because other parameters might not be equally distributed across different subgroups. Nevertheless, it is important to bear in mind that the presence of comorbidity or sleep medication use may be a possible confounder. Last, given the heterogeneous and often lacking reporting of negative effects^110^, we decided against analyzing negative effects in this study.

Currently, CBT-I is hardly available in the health care systems. Alternative CBT-I settings (in particular self-help settings such as iCBT-I and guided bibliotherapy) can help to enhance the scalability of CBT-I. Medium to large effect sizes for iCBT-I and guided bibliotherapy indicate that these self-help settings may be a viable alternative when synchronous communication settings are not available. Therefore, self-help interventions can complement care and reach patients who would otherwise not receive CBT-I or refuse onsite treatment. This study did not provide evidence for the superiority of any settings. However, synchronous communication settings (i.e., F2F, group, and telehealth) yielded the largest effect sizes. Given the large effect size and the strong evidence base for F2F, the latter should be considered as first-line treatment. Hence, it is essential to improve the structure of onsite care accordingly as the first and foremost task for improved global insomnia-related health.

## Supplementary Information

Supplementary Information.

## Data Availability

All data generated during this study are included in this published article (and its online supplementary materials).
