# Author Correction: Comparative efficacy of onsite, digital, and other settings for cognitive behavioral therapy for insomnia: a systematic review and network meta-analysis

**DOI:** 10.1038/s41598-025-99375-0

**Published:** 2025-05-14

**Authors:** Laura Simon, Lisa Steinmetz, Bernd Feige, Fee Benz, Kai Spiegelhalder, Harald Baumeister

**Affiliations:** 1 Department of Clinical Psychology and Psychotherapy, Institute of Psychology and Education, University Ulm, Lise-Meitner-Str. 16, 89081 Ulm, Germany; 2 Department of Psychiatry and Psychotherapy, Medical Center–University of Freiburg, Faculty of Medicine, University of Freiburg, Freiburg, Germany

Correction to: *Scientific Reports* 10.1038/s41598-023-28853-0, published online 02 February 2023

The original version of this Article contained an error in the data analysis for insomnia severity. Specifically, the Sleep Condition Indicator (SCI) scores were not inverted as required, affecting two of the included studies. Consequently, in the Abstract,

“For the primary outcome insomnia severity, all examined CBT-I settings except smartphone-delivered CBT-I yielded significant effects when compared to WL. Large standardized mean differences were found for individual onsite CBT-I (− 1.27;95%CI − 1.70, − 0.84), group-delivered CBT-I (− 1.00;95%CI − 1.42. − 0.59), telehealth (− 1.28;95%CI − 2.06, − 0.50), and guided bibliotherapy (− 0.99;95%CI − 1.67, − 0.32). Both guided iCBT-I (− 0.71;95%CI − 1.18, − 0.24) and unguided iCBT-I (− 0.78;95%CI − 1.18, − 0.38) yielded medium effect sizes.”

now reads:

“For the primary outcome insomnia severity, all examined CBT-I settings yielded significant effects when compared to WL. Large standardized mean differences were found for telehealth (−1.35;95%CI−1.73,−0.97), individual onsite CBT-I (−1.30;95%CI−1.51,−1.09), guided bibliotherapy (−1.05;95%CI−1.38,−0.71), smartphone (−1.04;95%CI−1.62,−0.46), group-delivered CBT-I (−1.01;95%CI−1.21,−0.82), and unguided iCBT-I (−1.01;95%CI−1.20,−0.82). Guided iCBT-I (−0.73;95%CI−0.95,−0.51) and unguided bibliotherapy (−0.67;95%CI−1.00,−0.35) yielded medium effect sizes.”

In the Results section,

“Results indicated significant effects of all examined CBT-I settings except smartphone. Neither of the CBT-I settings was superior to another. Large effect sizes were found for F2F (− 1.27, 95%CI − 1.70 to − 0.84), group (− 1.00, 95%CI − 1.42 to − 0.59), telehealth (− 1.28, 95%CI − 2.06 to − 0.50), and guided bibliotherapy (− 0.99, 95%CI − 1.67 to − 0.32). Both guided iCBT-I (− 0.71, 95% CI − 1.18 to − 0.24) and unguided iCBT-I (− 0.78, 95%CI − 1.18 to − 0.38) yielded medium effect sizes. P-Scores were the largest for F2F and telehealth (0.88 and 0.84, respectively; Supplementary Appendix S3). Substantial heterogeneity and inconsistencies were found (I^2^ = 95.5%; Q_withindesigns_ = 733.90, p < 0.0001; Q_betweendesigns_ = 210.72, p < 0.0001).”

now reads:

“Results indicated significant effects of all examined CBT-I settings. Neither of the CBT-I settings was superior to another. Large effect sizes were found for telehealth (−1.35, 95%CI −1.73 to −0.97), F2F (−1.30, 95%CI −1.51 to −1.09), guided bibliotherapy (−1.05, 95%CI −1.38 to −0.71), smartphone (−1.04, 95%CI −1.62 to −0.46), group (−1.01, 95%CI −1.21 to −0.82), and unguided iCBT-I (−1.01, 95%CI −1.20 to −0.82). Both guided iCBT-I (−0.73, 95% CI −0.95 to −0.51) and unguided bibliotherapy (−0.67, 95%CI −1.00 to −0.35) yielded medium effect sizes. P-Scores were the largest for telehealth and F2F (0.94 and 0.92, respectively; Supplementary Appendix S3). Substantial heterogeneity and inconsistencies were found (I^2^ = 77.7%; Q_withindesigns_ = 42.12, p = .0006; Q_betweendesigns_ = 150.12, p < .0001).”

In addition, Figure 3 in the Results section was incorrect. The original Figure 3 and accompanying legend appear below.

**Fig. 3:**
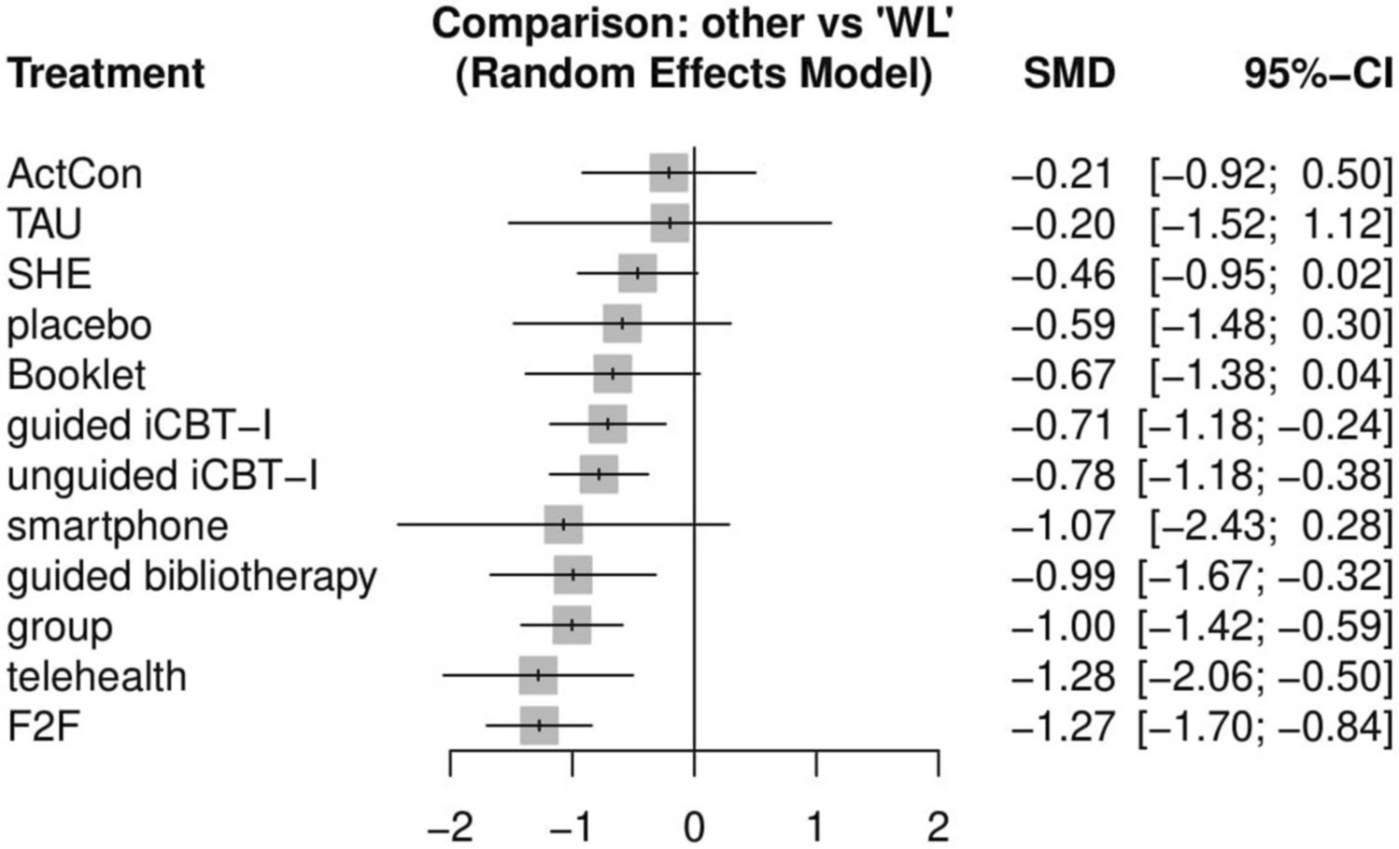
Forest plot insomnia severity. Treatments were ranked according to their P-Score.

In the Discussion section,

“For the primary outcome insomnia severity, large effect sizes were found for individual onsite CBT-I, group-delivered CBT-I, telehealth, and guided bibliotherapy. Both guided iCBT-I and unguided iCBT-I yielded medium effect sizes.”

now reads:

“For the primary outcome insomnia severity, large effect sizes were found for telehealth, F2F, guided bibliotherapy, smartphone, group-delivered CBT-I, and unguided iCBT-I. Guided iCBT-I and unguided bibliotherapy yielded medium effect sizes.”

“For bibliotherapy, guidance appears to be a crucial factor: whereas guided bibliotherapy achieved a large effect size, unguided bibliotherapy did not significantly differ from WL.”

now reads:

“Guidance might be an important factor in bibliotherapy, as guided bibliotherapy demonstrated a larger effect size and higher P-score compared to unguided bibliotherapy.”

“In line with the literature, medium effect sizes were found for most subjective sleep-related outcomes for guided and unguided iCBT-I. Interestingly, unguided iCBT-I did not differ from guided iCBT-I for most outcomes.”

now reads:

“In line with the literature, medium to large effect sizes were found for most subjective sleep-related outcomes for guided and unguided iCBT-I. Interestingly, unguided iCBT-I was comparable to guided iCBT-I for most outcomes and achieved a large effect size for insomnia severity, whereas guided iCBT-I yielded a medium effect size.”

“Nevertheless, given the medium effect sizes, iCBT-I, and in particular unguided iCBT-I, could have a major impact on the diminishment of the treatment gap and thus reach patients who would currently not receive any CBT-I.”

now reads:

“Nevertheless, given the medium to large effect sizes, iCBT-I, and in particular unguided iCBT-I, could have a major impact on the diminishment of the treatment gap and thus reach patients who would currently not receive any CBT-I.”

In the Supplemental Information, the P-Scores, net heat plot, and Funnel Plot for the outcome insomnia severity were incorrect. The original Supplementary Information file is provided below.

The original Article and accompanying Supplementary Information file have been corrected.

## Supplementary Information

Supplementary Information.

