# Supplementary material for: Comparative efficacy of onsite, digital, and other settings for cognitive behavioral therapy for insomnia: a systematic review and network meta-analysis
Source: Sci Rep. 2023 Feb 2;13:1929. doi: 10.1038/s41598-023-28853-0 (PMC9894949; doi:10.1038/s41598-023-28853-0)

Online Supplementary Materials

Comparative efficacy of onsite, digital, and other settings for cognitive behavioral therapy for insomnia: a systematic review and network meta-analysis

Simon L., Steinmetz L., Feige B., Benz F., Spiegelhalder K., Baumeister H.

Supplementary Appendix S1. PRISMA for network meta-analyses 2
Supplementary Appendix S2. Search string 6
Supplementary Appendix S3. P-Scores 7
Supplementary Table S1. Primary and secondary outcomes.. 10
Supplementary Table S2. Study characteristics 11
Supplementary Figure S1. Network plots and forest plots secondary outcomes............................ 18
Supplementary Figure S2. Net heat plots 21
Supplementary Figure S3. Risk of Bias- summary figure.. 35
Supplementary Figure S4. Risk of Bias- detailed figure.. 36
Supplementary Figures S5. Comparison-adjusted funnel plots....................................... 37

Supplementary Appendix S1. PRISMA for network meta-analyses

Section/Topic	Item #	Checklist Item	Reported on Page #
**TITLE**			
Title	1	Identify the report as a systematic review *incorporating a network meta-analysis (or related form of meta-analysis).*	1
**ABSTRACT**			
Structured summary	2	Provide a structured summary including, as applicable:<br>**Background:** main objectives<br>**Methods:** data sources; study eligibility criteria, participants, and interventions; study appraisal; and *synthesis methods, such as network meta-analysis.*<br>**Results:** number of studies and participants identified; summary estimates with corresponding confidence/credible intervals; *treatment rankings may also be discussed. Authors may choose to summarize pairwise comparisons against a chosen treatment included in their analyses for brevity.*<br>**Discussion/Conclusions:** limitations; conclusions and implications of findings.<br>**Other:** primary source of funding; systematic review registration number with registry name.	2
**INTRODUCTION**			
Rationale	3	Describe the rationale for the review in the context of what is already known*, including mention of why a network meta-analysis has been conducted.*	3-4
Objectives	4	Provide an explicit statement of questions being addressed, with reference to participants, interventions, comparisons, outcomes, and study design (PICOS).	4
**METHODS**			
Protocol and registration	5	Indicate whether a review protocol exists and if and where it can be accessed (e.g., Web address); and, if available, provide registration information, including registration number.	8
Eligibility criteria	6	Specify study characteristics (e.g., PICOS, length of follow-up) and report characteristics (e.g., years considered, language, publication status) used as criteria for eligibility, giving rationale. *Clearly describe eligible treatments included in the treatment network, and note whether any have been clustered or merged into the same node (with justification).*	5,7
Information sources	7	Describe all information sources (e.g., databases with dates of coverage, contact with study authors to	5

		identify additional studies) in the search and date last searched.	
Search	8	Present full electronic search strategy for at least one database, including any limits used, such that it could be repeated.	Supplementary Appendix S2
Study selection	9	State the process for selecting studies (i.e., screening, eligibility, included in systematic review, and, if applicable, included in the meta-analysis).	5-6
Data collection process	10	Describe method of data extraction from reports (e.g., piloted forms, independently, in duplicate) and any processes for obtaining and confirming data from investigators.	5
Data items	11	List and define all variables for which data were sought (e.g., PICOS, funding sources) and any assumptions and simplifications made.	5-6, Supplementary Table S1
**Geometry of the network**	**S1**	Describe methods used to explore the geometry of the treatment network under study and potential biases related to it. This should include how the evidence base has been graphically summarized for presentation, and what characteristics were compiled and used to describe the evidence base to readers.	6-7
Risk of bias within individual studies	12	Describe methods used for assessing risk of bias of individual studies (including specification of whether this was done at the study or outcome level), and how this information is to be used in any data synthesis.	8
Summary measures	13	State the principal summary measures (e.g., risk ratio, difference in means). *Also describe the use of additional summary measures assessed, such as treatment rankings and surface under the cumulative ranking curve (SUCRA) values, as well as modified approaches used to present summary findings from meta-analyses.*	6
Planned methods of analysis	14	Describe the methods of handling data and combining results of studies for each network meta-analysis. This should include, but not be limited to:<br>• *Handling of multi-arm trials;*<br>• *Selection of variance structure;*<br>• *Selection of prior distributions in Bayesian analyses; and*<br>• *Assessment of model fit.*	6
**Assessment of Inconsistency**	**S2**	Describe the statistical methods used to evaluate the agreement of direct and indirect evidence in the treatment network(s) studied. Describe efforts taken to address its presence when found.	8
Risk of bias across studies	15	Specify any assessment of risk of bias that may affect the cumulative evidence (e.g., publication bias, selective reporting within studies).	8
Additional analyses	16	Describe methods of additional analyses if done, indicating which were pre-specified. This may include, but not be limited to, the following:<br>• Sensitivity or subgroup analyses;<br>• Meta-regression analyses;	*no additional analyses were conducted*

		• *Alternative formulations of the treatment network; and*<br>• *Use of alternative prior distributions for Bayesian analyses (if applicable).*	
**RESULTS†**			
Study selection	17	Give numbers of studies screened, assessed for eligibility, and included in the review, with reasons for exclusions at each stage, ideally with a flow diagram.	9 (Figure 1)
**Presentation of network structure**	**S3**	Provide a network graph of the included studies to enable visualization of the geometry of the treatment network.	11 (Figure 2)
**Summary of network geometry**	**S4**	Provide a brief overview of characteristics of the treatment network. This may include commentary on the abundance of trials and randomized patients for the different interventions and pairwise comparisons in the network, gaps of evidence in the treatment network, and potential biases reflected by the network structure.	10
Study characteristics	18	For each study, present characteristics for which data were extracted (e.g., study size, PICOS, follow-up period) and provide the citations.	Supplementary Table S2
Risk of bias within studies	19	Present data on risk of bias of each study and, if available, any outcome level assessment.	15, Supplementary Figures S3 and S4
Results of individual studies	20	For all outcomes considered (benefits or harms), present, for each study: 1) simple summary data for each intervention group, and 2) effect estimates and confidence intervals. *Modified approaches may be needed to deal with information from larger networks.*	10-15
Synthesis of results	21	Present results of each meta-analysis done, including confidence/credible intervals. *In larger networks, authors may focus on comparisons versus a particular comparator (e.g. placebo or standard care), with full findings presented in an appendix. League tables and forest plots may be considered to summarize pairwise comparisons.* If additional summary measures were explored (such as treatment rankings), these should also be presented.	10-15
**Exploration for inconsistency**	**S5**	Describe results from investigations of inconsistency. This may include such information as measures of model fit to compare consistency and inconsistency models, *P* values from statistical tests, or summary of inconsistency estimates from different parts of the treatment network.	10-15, Supplementary Figure S2
Risk of bias across studies	22	Present results of any assessment of risk of bias across studies for the evidence base being studied.	15, Supplementary Figure S5
Results of additional	23	Give results of additional analyses, if done (e.g., sensitivity or subgroup analyses, meta-regression	*no additional analyses were*

analyses		analyses*, alternative network geometries studied, alternative choice of prior distributions for Bayesian analyses,* and so forth).	*conducted*
**DISCUSSION**			
Summary of evidence	24	Summarize the main findings, including the strength of evidence for each main outcome; consider their relevance to key groups (e.g., healthcare providers, users, and policy-makers).	15-17
Limitations	25	Discuss limitations at study and outcome level (e.g., risk of bias), and at review level (e.g., incomplete retrieval of identified research, reporting bias). *Comment on the validity of the assumptions, such as transitivity and consistency. Comment on any concerns regarding network geometry (e.g., avoidance of certain comparisons).*	17-18
Conclusions	26	Provide a general interpretation of the results in the context of other evidence, and implications for future research.	18-19
**FUNDING**			
Funding	27	Describe sources of funding for the systematic review and other support (e.g., supply of data); role of funders for the systematic review. This should also include information regarding whether funding has been received from manufacturers of treatments in the network and/or whether some of the authors are content experts with professional conflicts of interest that could affect use of treatments in the network.	8

From: Hutton, B. *et al.* The PRISMA Extension Statement for Reporting of Systematic Reviews Incorporating Network Meta-analyses of Health Care Interventions: Checklist and Explanations. *Ann. Intern. Med.* **162**, 777–784 (2015).

Supplementary Appendix S2. Search string

The search strings were developed by combining terms indicative of insomnia, CBT-I, and CBT-I components. The databases PsycINFO, PsycARTICLES, MEDLINE, PubMed, and CINAHL were searched for publications from 1987, which is the publication date of DSM-III-R (American Psychiatric Association, 1987), until November 23rd, 2021.

Pubmed | results by year 1987

(cognitive therapy[Title/Abstract] OR cognitive behavio* therapy[Title/Abstract] OR behavio* therapy[Title/Abstract] OR sleep behavior therapy[Title/Abstract] OR CBT[Title/Abstract] OR CBT-I[Title/Abstract] OR paradoxical intention[Title/Abstract] OR paradoxical techniques[Title/Abstract] OR cognitive control[Title/Abstract] OR cognitive reconstr*[Title/Abstract] OR cognitive restruct*[Title/Abstract] OR cognitive refocusing[Title/Abstract] OR imagery[Title/Abstract] OR problem solving[Title/Abstract] OR constructive worry[Title/Abstract] OR stimulus control[Title/Abstract] OR sleep restriction[Title/Abstract] OR sleep hygiene[Title/Abstract] OR psychoeducation[Title/Abstract] OR relaxation[Title/Abstract])

AND

(insomni*[Title/Abstract] OR sleep initiation[Title/Abstract] OR sleep maintenance[Title/Abstract])

Medline, **PsychInfo**, **Cinahl**, **PsycArticles** searched via Ebsco Host | Published Date: from 1987, Field: AB Abtract

("cognitive therapy" OR "cognitive behavio* therapy" OR "behavio* therapy" OR "sleep behavior therapy" OR "CBT-I" OR "CBT" OR "paradoxical intention" OR "paradoxical techniques" OR "cognitive control" OR "cognitive reconstr*" OR "cognitive restruct*" OR "cognitive refocusing" OR "imagery" OR "problem solving" OR "constructive worry" OR "stimulus control" OR "sleep restriction" OR "sleep hygiene" OR "psychoeducation" OR "relaxation")

AND

"insomni*" or "sleep initiation" or "sleep maintenance"

Supplementary Appendix S3. P-Scores

**P-Scores: Insomnia severity**	
Telehealth	0.94
F2F	0.92
Guided bibliotherapy	0.73
Smartphone	0.71
Group	0.70
Unguided iCBT-I	0.70
Guided iCBT-I	0.47
Unguided bibliotherapy	0.43
TAU	0.26
SHE	0.26
Placebo	0.21
Active contact control	0.16
WL	0.01

**P-Scores: Subjective sleep quality**	
Guided bibliotherapy	0.83
F2F	0.82
Group	0.78
Smartphone	0.74
Telehealth	0.71
Guided iCBT-I	0.63
Unguided iCBT-I	0.49
Unguided bibliotherapy	0.45
TAU	0.38
Placebo	0.30
SHE	0.18
Active contact control	0.14
WL	0.14

**P-Scores: Subjective total sleep time**	
Unguided bibliotherapy	0.89
Group	0.88
Guided iCBT-I	0.75
Guided bibliotherapy	0.71
TAU	0.61
F2F	0.48
Unguided iCBT-I	0.45
Telehealth	0.40
SHE	0.29
WL	0.19
Active contact control	0.17
Placebo	0.17

**P-Scores: Subjective sleep efficiency**	
Group	0.96
Guided bibliotherapy	0.83
F2F	0.68
Telehealth	0.66
Guided iCBT-I	0.59
TAU	0.58
Unguided iCBT-I	0.57
Unguided bibliotherapy	0.51
WL	0.21
Placebo	0.17
Active contact control	0.16
SHE	0.08

**P-Scores: Subjective sleep onset latency**	
Guided bibliotherapy	0.92
Telehealth	0.87
Group	0.81
F2F	0.81
Guided iCBT-I	0.60
Unguided iCBT-I	0.54
Unguided bibliotherapy	0.53
WL	0.27
TAU	0.26
SHE	0.24
Active contact control	0.10
Placebo	0.05

**P-Scores: Subjective wake after sleep onset**	
Group	0.91
Telehealth	0.83
F2F	0.72
Guided bibliotherapy	0.67
Unguided iCBT-I	0.62
Guided iCBT-I	0.59
TAU	0.55
Unguided bibliotherapy	0.48
Placebo	0.26
WL	0.16
SHE	0.14
Active contact control	0.09

**P-Scores: Objective total sleep time**	
WL	0.94
Telehealth	0.85
Active contact control	0.72
F2F	0.45
SHE	0.43
Unguided iCBT-I	0.32
Group	0.16
Placebo	0.13

**P-Scores: Objective sleep efficiency**	
Telehealth	0.85
F2F	0.77
SHE	0.59
Group	0.53
Unguided iCBT-I	0.49
Active contact control	0.43
WL	0.32
Placebo	0.29
TAU	0.23

**P-Scores: Objective sleep onset latency**	
Telehealth	0.86
F2F	0.82
SHE	0.68
Active contact control	0.55
Unguided iCBT-I	0.42
TAU	0.30
Group	0.28
WL	0.09

**P-Scores: Objective wake after sleep onset**	
Group	0.83
F2F	0.63
Unguided iCBT-I	0.61
SHE	0.58
TAU	0.45
WL	0.43
Placebo	0.27
Active contact control	0.19

**P-Scores: Response rates**	
Guided bibliotherapy	0.82
Unguided iCBT-I	0.73
Telehealth	0.69
F2F	0.64
Unguided bibliotherapy	0.47
Group	0.46
SHE	0.41
Guided iCBT-I	0.26
WL	0.03

**P-Scores: Remission rates**	
Telehealth	0.89
F2F	0.85
Guided bibliotherapy	0.80
Unguided iCBT-I	0.57
Unguided bibliotherapy	0.47
Group	0.35
Guided iCBT-I	0.25
SHE	0.19
WL	0.12

**P-Scores: Intervention completion rates**	
Group	0.83
Unguided iCBT-I	0.7
Guided iCBT-I	0.62
F2F	0.58
SHE	0.55
Placebo	0.54
Smartphone	0.54
Guided bibliotherapy	0.45
TAU	0.43
Telehealth	0.42
Active contact control	0.37
Unguided bibliotherapy	0.3
WL	0.18

Supplementary Table S1. Primary and secondary outcomes

Outcome	Measured via	Order[1]	Effect size
**Primary outcome**			
Insomnia severity	self-report questionnaires (e.g., Insomnia Severity Index ISI); (Célyne H. Bastien et al., 2001))	ISI, other self-report questionnaires	Standardized mean difference (*SMD*)
**Secondary outcomes**			
Subjective sleep quality	sleep diaries or self-report questionnaires (e.g., Pittsburgh Sleep Quality Index (PSQI); (Buysse et al., 1989)	PSQI, sleep diary, other self-report questionnaires	*SMD*
Subjective total sleep Time	sleep diaries or self-report questionnaires	sleep diary, PSQI	*SMD*
Subjective sleep efficiency	sleep diaries or self-report questionnaires	sleep diary, PSQI	*SMD*
Subjective sleep onset latency	sleep diaries or self-report questionnaires	sleep diary, PSQI	*SMD*
Subjective wake after sleep onset	sleep diaries or self-report questionnaires	sleep diary, PSQI	*SMD*
Objective total sleep time	polysomnography or actigraphy	polysomnography, accelerometer	*SMD*
Objective sleep efficiency	polysomnography or actigraphy	polysomnography, accelerometer	*SMD*
Objective sleep onset latency	polysomnography or actigraphy	polysomnography, accelerometer	*SMD*
Objective wake after sleep onset	polysomnography or actigraphy	polysomnography, accelerometer	*SMD*
Response (Clinically Significant Improvement)	pre-defined criterion pre-to-post-treatment (e.g., ≥8-point drop of the ISI score)	ISI point-drop=≥8, SE increase ≥ 10%, other	SMD (transformed through a Freeman-Tukey double arcsine transformation)
Remission	pre-defined criterion post-treatment (e.g., ISI score<7)	ISI score<7, SE≥85%, SOL<31, WASO<31, Other	SMD (transformed through a Freeman-Tukey double arcsine transformation)
Intervention completion	Percentage of participants completing the intervention[2]		SMD (transformed through a Freeman-Tukey double arcsine transformation)

Note.[1]The order of the data sources depicts which data was preferred if there were several data for the construct. [2] Either as defined by authors or as depicted in the flowchart

Supplementary Table S2. Study characteristics

Study	Insomnia definition according to	N [Pre-Post]	Inclusion sleep comorbidity	Inclusion comorbidity	Use of sleep medication	Outcomes	Setting	Komponenten	Number of sessions	Duration of sessions	Therapist specialization	n [Pre-Post]	Age [Years: Mean (SD)]	Sex [% Female]	Mean insomnia duration per group
Alessi et al. 2016	ICSD-2	159 - 150	Partially excluded	Partially excluded	No restriction	ISI, PSQI, SOL_diary, WASO_diary, SE_diary	F2F	SHE, SR, SK, Cog	5	60 minutes	Sleep coaches	54 - 52	72.1 (7.9)x	3.8x	NA
							Group	SHE, SR, SK, Cog	5	60 minutes	Sleep coaches	52 - 45*	72.1 (7.9)x	3.8x	NA
							SHE	SHE	5	60 minutes	Sleep coaches	53 - 53	72.4 (7.3)	2.9	NA
Arnedt et al. 2013	RDC	30 - 29	Excluded	Partially excluded	Required to be stable	ISI, PSQI, SOL_diary, WASO_diary, SE_diary, TST_diary, SQ_diary, Rem, Res, Comp	Guided bibliotherapy	SHE, SR, SK, Cog_restruct	4 to 8	15 - 60-minutes (contingent on treatment response)	Psychologist	18 - 15	38.1 (14.6)	100	9.2 (10.8)
							SHE	SHE	1	15 to 20minutes telephone session with a study therapist	Psychologist	15 - 14	40 (14.6)	80	8.1 (10.9)
Arnedt et al. 2021	ICSD-3	65 - 65	Excluded	Partially excluded	No restriction	ISI, SOL_diary, WASO_diary, SE_diary, TST_diary, Rem, Res, Comp	Telehealth	SHE, SR, SK, Cog_control, R	6	30 to 60 minutes	Sleep Medicine Expert	33 - 33	43.7 (17.4)	69.7	NA
							F2F	SHE, SR, SK, Cog_control, R	6	30 to 60 minutes	Sleep Medicine Expert	32 - 32	50.9 (14.5)	71.9	NA
Ballesio et al. 2018	DSM-5	10 - 10	Not reported	Not reported	Not reported	ISI, SOL_diary, SE_diary, Comp	F2F	SHE, SR, SK, Cog_restruct, R	4		Psychologist	6 - 6	22.8 (4.29)x	90x	NA
							Unguided bibliotherapy	SHE, SR, SK, Cog_restruct, R				4 - 4	22.8 (4.29)x	90x	NA
Bastien at al. 2004	DSM-IV and ICSD	45 - 45	Excluded	Partially excluded	Not allowed	ISI, SOL_diary, WASO_diary, TST_diary, SE_diary, SQ_diary, Rem, Comp	Group	SHE, SR, SK, Cog_restruct	8	90 minutes (4 – 6 persons per group)	Psychologist	16 - 16	40 (10.35)	68.75x	15.35 (14.13)
							F2F	SHE, SR, SK, Cog_restruct	8	50 minutes	Psychologist	15 - 15	43.8 (9.98)	73.33x	15.64 (11.73)
							Guided bibliotherapy	SHE, SR, SK, Cog_restruct	8	20 minutes	Psychologist	14 - 14	41.64 (9.49)	50x	14.71 (10.2)
Blom et al. 2015	DSM-5	48 - 45*	Excluded	Partially excluded	No restriction	ISI, SOL,_diary, TST_diary, SE_diary, SQ-diary, Rem, Res, Comp	Group	SHE, SR, SK, Cog_restruct, R	8	120 minutes	Psychologist	24 - 23*	52.6 (16.6)	62.5	NA
							Guided iCBT-I	SHE, SR, SK, Cog_restruct, R	8		Psychologist	24 - 22*	56.1 (10.2)	33	NA
Bothelius et al. 2013	RDC	66 - 54	Excluded	Partially excluded	No restriction	ISI, SOL_diary, WASO_diary, Rem, Comp	Group	SHE, SR, SK, Cog_restruct, Cog_PI, R_PMR	5	60 to 90 minutes	Nurse	32* - 26	48.1 (13.2)	78	NA
							WL					34* - 28	53 (9.4)	94	NA

Cheng et al. 2019	DSM-5	1385 - 658	Excluded	Partially excluded	Not reported	ISI, Rem, Res	Unguided iCBT-I	SHE, SR, SK, Cog_Restruct, Cog_PI, R_PMR, R_auto, R_mindful	6		Virtual therapist	946 - 358	44.5 (15.8)	78	NA
							SHE	SHE	6		None	439 - 300	45.7 (15.1)	80	NA
Drake et al. 2019	DSM-5	154 - 150	Excluded	Partially excluded	Not allowed	ISI, SOL_diary, WASO_diary, SE_diary, TST_diary, SQ_diary, Rem, Comp	F2F	SHE, SR, SK, Cog_Restruct, Cog_PI, R_PMR, R_auto	6		Nurse	52 - 50	55.32 (5.9)	100	NA
							SHE	SHE			Nurse	50 - 50	57.24 (5.55)	100	NA
Edinger et al. 2001	DSM-III	75 - 70	Partially excluded	Partially excluded	Not allowed	ISQ, SOL_diary, WASO_diary, SE_diary, TST_diary, SQ_diary, Res, Comp	F2F	SHE, SR, SK, Cog_Restruct	6	30 to 60 minutes	Psychologist	25 - 23	55.8 (12.1)	44	13 (12.2)
							Placebo	Placebo	6	30 to 60 minutes	Psychologist	25 - 24	55.7 (9.5)	52	14.8 (11.5)
Edinger et al. 2007	DSM-IV	35* - 30*	Excluded	Partially excluded	Not allowed	ISQ, SOL_diary, WASO_diary, SE_diary, TST_diary, Rem, Res, Comp	F2F	SHE, SR, SK, Cog_Restruct	4	45 to 60 minutes (first session), 15-30 minutes (other sessions)	Psychologist	24* - 21*	57 (10.2)	50	NA
							ActCon	Active control				11* - 9	52.4 (7.3)	36.36	NA
Edinger et al. 2009	DSM-IV and RDC	81* - 69*	Partially excluded	Partially excluded	No restriction	ISQ, PSQI, SOL_diary, WASO_diary, SE_diary, TST_diary, SOL_act, WASO_act, SE_act, TST_act, Rem, Comp	F2F	SHE, SR, SK, Cog_Restruct	4	30 to 60 minutes	Psychologist	41* - 36*	54.39 (NA)	14.63	11.51 (NA)
							SHE	SHE	4	30 to 60 minutes	Psychologist	40* - 33*	54 (NA)	12.5	9 (NA)
Espie et al. 2001	ICSD	139 - 139	Partially excluded	Partially excluded	No restriction	SOL_diary, WASO_diary, TST_diary, Comp	Group	SHE, SK, SR, R, Cog_restruct	6	50 minutes	Nurse	74only post reported - 74	51.4 (17.1)x	68.34x	NA
							ActCon	Active control				65only post reported - 65	51.4 (17.1)x	68.34x	NA
Espie et al. 2007	DSM-IV and ICSD-R	201 - 178	Partially excluded	Partially excluded	No restriction	PSQI, SOL_diary, WASO_diary, SE_diary, TST_diary, SOL_act, WASO_act, SE_act, Rem, Comp	Group	SHE, SR, SK, R, Cog_restruct	5	60 minutes	Nurses	107 - 95	54.4 (15.4)	67.29	11.6 (9.79)
							TAU	TAU				94 - 83	54.1 (14.4)	69.15	10.6 (12.2)
Espie et al. 2012	DSM-5	164 - 131	Excluded	Excluded	No restriction	SCI, SOL_diary, WASO_diary, SE_diary, TST_diary, SQ_diary, Rem, Comp	Unguided iCBT-I	SHE, SR, SK, Cog_Restruct, Cog_PI, R_PMR, R_auto, R_mindful	6		Virtual therapist	55 - 43	50.7 (13.8)	72.7	NA
							Placebo	Placebo	6		Virtual therapist	55 - 41	47.3 (13)	76.4	NA
							WL					55 - 47	49.1 (13.7)	70.4	NA

Espie et al. 2019	DSM-5	1711 - 985	Excluded	Partially excluded	No restriction	SCI, SOL_diary, WASO_diary, SE_diary, TST_diary, SQ_diary, Comp	Unguided iCBT-I	SHE, SR, SK, Cog_restruct, Cog_PI, R_PMR, R_auto, R_mindful	6		Virtual therapist	853 - 468	48.4 (13.9)	76.7	NA
							SHE	SHE				858 - 517	47.7 (13.6)	78.7	NA
Freeman et al. 2017	SCI ≤16	3755 - 1875	Not reported	No restriction	Not reported	SCI, Rem, Comp	Unguided iCBT-I	SHE, SR, SK, Cog_restruct, Cog_PI, R_PMR, R_auto, R_mindful	6		Virtual therapist	1891 - 733	24.8 (7.7)	72	NA
							TAU					1864 - 1142	24.6 (7.6)	71	NA
Gehrman et al. 2021	DSM-5	60 - 54	Excluded	Partially excluded	Not allowed	ISI, Comp	F2F	SHE, SR, SK, Cog, R	6 to 8		Psychologist / Students	20 - 24	33.7 (10.58)	65	NA
							Telehealth	SHE, SR, SK, Cog, R				21 - 26	33.14 (10)	62	NA
							WL					19 - 17	31.21 (8.7)	68	MA
Gieselmann & Pietrowsky 2019	DSM-5	72 - 66	Excluded	Partially excluded	Required to be stable	PSQI, SOL_diary, SE_diary, TST_diary, SOL_act, SE_act, TST_act, Rem, Comp	F2F	SHE, SR, R_image	3		Psychologist / students	27 - 24	39.3 (14.47)	48	9.16 (10.17)
							Telehealth	SHE, SR, R_image	3		Psychologist / students	23 - 23	39.47 (11.16)	56	7.12 (5.61)
							WL					22 - 19	42.74 (11.73)	53	12.09 (10.52)
Hagatun et al. 2018	DSM-IV	181 - 142	Partially excluded	Partially excluded	No restriction	ISI, SOL_diary, WASO_diary, SE_diary, TST_diary, Rem, Comp	Unguided iCBT-I	SHE, SR, SK, Cog_restruct	6			95 - 77	45 (12.4)	64	categorical scale
							SHE	SHE				86 - 65	44.8 (13.7)	71	categorical scale
Ho et al. 2014	difficulty initiating or maintaining sleep, early morning awakening or non-restorative sleep with associated distress or impairment for three or more nights per week for at least three months	312 - 184	Not reported	Partially excluded	Not reported	ISI, PSQI, SOL_diary, WASO_diary, SE_diary, TST_diary, SQ_diary, Rem, Comp	Unguided iCBT-I	SHE, SR, SK, Cog_restruct, Cog_control, R	6			104 - 61*	38.6 (11.8)	67.31	8 (8.9)
							Guided iCBT-I	SHE, SR, SK, Cog_restruct, Cog_control, R	6		Psychologist	103 - 58	36.9 (13)	70.87	8 (7.8)
							WL					105 - 65	39.9 (12.7)	75.24	9.4 (8.9)
Ho et al. 2021	DSM-5 and SCI	25 - 15	Not reported	Partially excluded	Not allowed	ISI, Rem	Group	SHE, SR, SK, Cog_Restruct, Cog_control, R_PMR	6	120 minutes	Psychologist / Students	13 - 7	43.1 (14.3)	84.62	15 (12.9)
							WL					13 - 8	36.6 (11.7)	69.23	12.7 (12.5)
Holmqvist et al. 2014	RDC	73 - 54	Partially excluded	Partially excluded	No restriction	ISI, SOL_diary, WASO_diary, SE_diary, TST_diary, SQ_diary, Rem, Res	Telehealth	SHE, SR, SK, Cog_control, Cog_problem, R_PMR, R_image	6		Sleep medicine experts	34 - 28	NA (NA)	79.4	NA
							Unguided iCBT-I	SHE, SR, SK, Cog_control, Cog_problem, R_PMR, R_image	6			39 - 26	NA (NA)	71.8	NA

Irwin et al. 2014	DSM-IV and ICSD-2	75 - 72	Partially excluded	Partially excluded	Not allowed	AIS, PSQI, SOL_diary, WASO_diary, SE_diary, TST_diary	Group	SHE, SR, SK, Cog, R	16	120 minutes	Therapists	50 - 48	66.4 (6.1)	78	NA
							SHE	SHE	16	120 minutes	Therapists	25 - 24	66.4 (7.7)	72	NA
Jernelov et al. 2012	RDC	133 - 126*	Partially excluded	Partially excluded	No restriction	ISI, SOL_diary, WASO_diary, SE_diary, TST_diary, SQ_diary, Rem, Res, Comp	Guided bibliotherapy	SHE, SR, SK, Cog_Restruct, R	6		Psychologist	44 - 43	50.8 (11.8)	75	18.2 (3.5)
							Unguided bibliotherapy	SHE, SR, SK, Cog_Restruct, R	6			45 - 44	47.4 (13.3)	80	18.3 (3.3)
							WL					44* - 39	45.4 (16)	90.9	18.3 (3.3)
Johann et al.2020	DSM-5	46 - 44	Excluded	Partially excluded	Not allowed	ISI, TST_diary, SOL_diary, WASO_diary, SE_diary	F2F	SHE, SR, Cog_restruct, Cog_control, Cog_PI, R_PMR, R_Auto	8	50 minutes	Psychologist	23 - 23	40.8 (14)	60.87	NA
							WL					23 - 21	41.2 (15.1)	65.22	NA
Kallestad et al. 2021	DSM-5	101 - 94*	Excluded	Partially	No restriction	ISI, SOL_diary, WASO_diary, TST_diary, SE_diary, Rem, Comp	F2F	SHE, SR, SK, Cog_Restruct	3 to 8		Psychologist	52 - 50*	41.3 (12.5)		13 (10.4)
							Unguided iCBT-I	SHE, SR, SK, Cog_Restruct	6	45 to 60 minutes		49 - 44*	41.4 (10.5)		12.6 (11.5)
Kaldo et al. 2020	RDC	40 - 37*	Excluded	Partially excluded	No restriction	SOL_diary, WASO_diary, TST_diary, SE_diary, SQ_diary, ISI, Rem, Res, Comp	Group	SHE, SR, SK, Cog, Cog_Restruct, R, R_mindful	6	45 to 85 minutes	Psychologist / Students	20 - 18*	52.2 (10.7)	65	17.2 (12.9)
							WL					20 - 15*	57.9 (10.8)	75	11.1 (12.3)
Krieger et al. 2019	ICSD-3	104 - 91	Excluded	Partially excluded	No restriction	ISI, PSQI, Rem, Res, Comp	Guided iCBT-I	SHE, SR, SK, Cog_restruct, R_PMR	8		Psychologist / Students	42 - 40*	42.17 (12.4)	61.9	NA
							WL					21 - 20	45.24 (12.4)	81	NA
Kyle et al. 2020	DSM-5	410 - 336	Excluded	Partially excluded	Not allowed	ISI, SE_PSQI, Comp	Unguided iCBT-I	SHE, SR, SK, Cog_restruct, Cog_PI, R_PMR, R_auto, R_mindful	6	15 to 20 minutes	Virtual Therapist	205 - 155	52.5 (11.2)	85.4	NA
							WL					205 - 181	52.2 (11.7)	87.7	NA
Lancee et al. 2012	DSM-IV	623 - 531	Partially excluded	Partially excluded	No restriction	SE_diary, TST_diary, SOL_diary, WASO_diary, Rem, Comp	Unguided bibliotherapy	SHE, SR, SK, Cog_Restruct, Cog_PI, R_PMR, R_Auto	6			203 - 179*	51.2 (12.8)	74.4	NA
							Guided iCBT-I	SHE, SR, SK, Cog_Restruct, Cog_PI, R_PMR, R_Auto	6			216 - 166*	52.2 (11.4)	68.7	NA
							WL					200 - 184*	51.9 (12.2)	68	NA

Lancee et al. 2015	DSM-5	63 - 54*	Partially excluded	Partially excluded	No restriction	SE_diary, TST_diary, SOL_diary, WASO_diary, SQ_diary, ISI, Rem, Res, Comp	Guided iCBT-I	SHE, SR, SK, Cog_Restruct, Cog_PI, R_PMR, R_Auto	6		Psychologist / Students	36 - 25	47.47 (14.37)	83.3	NA
							WL					27 - 18	49.98 (13.71)	74.1	NA
Lancee et al. 2016	DSM-5	90 - 81*	Partially excluded	Partially excluded	No restriction	SOL_diary, WASO_diary, TST_diary, SQ_diary, ISI, Rem, Res, Comp	F2F	SHE, SR, SK, Cog_restruct, R_PMR	6	45 minutes	Specialized Psychologist	30 - 29*	38.5 (13.1)	73.3	NA
							Guided iCBT-I	SHE, SR, SK, Cog_restruct, R_PMR	6		Psychologist / Students	30 - 26*	41.2 (14.1)	86.7	NA
							WL					30 - 26*	45.1 (13.7)	83.3	NA
Lopez et al. 2019	DSM-5	46 - 38	Excluded	Excluded	No restriction	TST_diary, SE_diary, SOL_diary, WASO_diary, ISI, Comp	Unguided iCBT-I	SHE, SR, SK, Cog_Restruct, R_Auto, R_mindful, R_PMR	7			23 - 18	46 (11)	82.61	11 (15)
		46 -					SHE	SHE	1	60 minutes		23 - 20	45 (13)	65.22	11 (14)
Lorenz et al. 2019	DSM-5	56 - 52	Excluded	Partially excluded	Required to be stable	ISI, Rem, Comp	Unguided iCBT-I	SHE, SR, SK, R_PMR, Cog_Restruct	6		Virtual therapist	29 - 25	41.72 (17.31)	72	NA
							WL					27 - 27	44.04 (20.05)	67	NA
Lovato et al. 2014	(1) WASO ≥30 min, at least three nights per week for at least 6 months, (2) impaired daytime functioning	118 - 109	Excluded	Partially excluded	Not allowed	SE_diary, SOL_diary, TST_diary, WASO_act, TST_act, SE_act, ISI, Rem, Comp	Group	SHE, SR, Cog_Restruct	4	60 minutes	Psychologist	86 - 78	63.76 (6.45)x	50x	NA
							WL					32 - 31	63.76 (6.45)x	50x	NA
Lovato et al. 2016	(1) WASO ≥30 min, at least three nights per week for at least 6 months, (2) impaired daytime functioning	91 - 82	Excluded	Partially excluded	Not allowed	ISI, SOL_diary, WASO_diary, SE_diary, TST_diary, SE_act, SOL_act, WASO_act, TST_act, Rem, Comp	roup	SHE, SR, Cog_Restruct	4	60 minutes	Therapists / Psychologists	63 - 57	62.94 (6.45)	50	NA
							WL					28 - 26	63.34 (6.41)	56.41	NA
Majd et al. 2020	DSM-5	312 - 295	Partially excluded	Partially excluded	Not reported	ISI, PSQI, Comp	Smartphone	SHE, SR, Cog_Restruct, Cog_probelm, R_image	6			156 - 147	36.21 (5.81)	53.9	1.3 (NA)
							SHE	SHE				156 - 148	35.29 (5.76)	57.7	1.3 (NA)
Mimeault & Morin 1999	ICSD and DSM-IV	58 - 54	Excluded	Partially excluded	No restriction	SII, SOL_diary, WASO_diary, SE_diary, TST_diary, SQ_diary, PSQI, Rem, Comp	Guided bibliotherapy	SHE, SK, SR, Cog_Restruct	6		Therapist	18 - 18	45.61 (8.49)	61.11	12.43 (10.22)
							ActCon	Active control				18 - 18	59.94 (13.43)	61.11	12.86 (8.98)
							Unguided bibliotherapy	SHE, SK, SR, Cog_Restruct	6			18 - 18	49.83 (13.26)	55.56	17.13 (11.09)

Morin et al. 1993	ICSD	24 - 24	Partially excluded	Partially excluded	No restriction	SOL_diary, WASO_diary, TST_diary, SE_diary, SOL_psg, WASO_psg, TST_psg, SE_psg, Comp	Group	SHE, SR, SK, Cog_Restruct	8	90 minutes	Psychologist	12 - 12	67.1 (5.3)x	70.8x	NA
							ActCon	Active control				10 - 10	67.1 (5.3)x	70.8x	NA
Ritterband et al. 2009	DSM-IV	45 - 43*	Excluded	Partially excluded	Required to be stable	ISI, SOL_diary, WASO_diary, SE_diary, Rem, Comp	Unguided iCBT-I	SHE, SR, SK, Cog_Restruct	6			22 - 21	44.68 (10.61)	81.82	10.14 (8.26)
							WL					22 - 22	45.05 (11.67)	72.73	11.04 (8.89)
Ritterband et al. 2017	DSM-5	303 - 275*	Excluded	Partially excluded	Required to be stable	ISI, SOL_diary, WASO_diary, SE_diary, TST_diary, SQ_diary, Rem, Comp	Unguided iCBT-I	SHE, SR, SK, Cog_Restruct	6			151 - 133*	43.75 (11.34)	68.2	15 (NA)
							SHE	SHE				152 - 142*	42.81 (11.86)	75.7	7 (NA)
Sandlund et al. 2017	DSM-IV	165 - 132	Excluded	Partially excluded	No restriction	ISI, SOL_diary, SE_diary, WASO_diary, TST_diary, SQ_diary, Rem, Res, Comp	Group	SHE, SK, SR, Cog_Restruct, Cog_PI, Cog_Control, Cog_problem, R	7	120 minutes	Nurses	82 - 72	55 (17.1)	71.1	12 (NA)
							WL					71 - 60	54 (17.4)	74.7	13 (NA)
Sato et al. 2019	DSM-5	23 - 22	Not reported	Partially excluded	No restriction	SOL_PSQI, SE_PSQI, TST_PSQI, PSQI, Rem, Comp	Guided iCBT-I	SHE, SK, SR, Cog_restruct, R, R_PMR	5		Therapist	11 - 11	49.4 (13.8)	81.3	6.3 (5.1)
							SHE	SHE	As part of the UC, both the UC only and ICBT plus UC groups received email magazines with general information about insomnia and hypnotics (in PDF format) by the research team 4 times over a 6 week period			12 - 12	50.5 (8.8)	75	6 (7.7)
Ström et al. 2004	DSM-IV and ICSD	109 - 83*	Excluded	Partially excluded	No restriction	SOL_diary, SE_diary, SQ_diary, Comp	Guided iCBT-I	SHE, SR, SK, Cog_Restruct, R	5		Psychologist	54 - 32*	46.2 (11.6)	66.67	12.9 (8.6)
							WL					55 - 51	43.9 (11.4)	62.75	9 (7.8)
Taylor et al. 2014	DSM-5	34 - 29	Excluded	Partially excluded	Not allowed	ISI, SE_diary, TST_diary, SOL_diary, WASO_diary, SQ_diary, PSQI, SE_act, TST_act, SOL_act, WASO_act, Comp	F2F	SHE, SR, SK, Cog_Restruct, R_PMR, R_auto	6			17 - 16	19.47 (1.66)	23.5	NA
							WL					17 - 13	19.94 (2.49)	58.8	NA
Taylor et al. 2017	DSM-5	100 - 86	Partially excluded	Partially excluded	Required to be stable	ISI, SE_diary, TST_diary, SOL_diary, WASO_diary, SQ_diary, Comp	F2F	SHE, SR, SK, Cog_Restruct, R	6		None	34 - 27	34.53 (8.27)	18	NA

							Unguided iCBT-I	SHE, SR, SK, Cog_Restruct, R	6	60 minutes	Psychologist / Students / Social worker	33 - 30	30.79 (6.42)	21	NA
							ActCon	Active control	in call every week			33 - 29	32.82 (8.11)	12	NA
Taylor et al. 2018	DSM-5	151 - 133	Partially excluded	Partially excluded	Required to be stable	ISI, SE_diary, TST_diary, SOL_diary, WASO_diary, SQ_diary, SE_act, TST_act, SOL_act, WASO_act , Comp	F2F	SHE, SR, SK, Cog_Restruct, R	6	60 minutes	Psychologist / Students / Social worker	42 - 30	32.21 (7.18)	18	NA
							ActCon	Active control	in call every week			43 - 36	32.67 (7.97)	17	NA
van der Zweerde et al. 2020	DSM-5	134 - 105*	Partially excluded	Partially excluded	No restriction	ISI, SOL_diary, SE_diary, WASO_diary, TST_diary, SQ_diary, Rem, Res, Comp	Guided iCBT-I	SHE, SR, SK, Cog_Restruct, R	5		Nurses	69 - 55	51.7 (15.77)	62	NA
							WL					65 - 50	49.4 (16.01)	68	NA
van Straten et al. 2014	DSM-IV	118 - 82*	Not reported	Partially excluded	No restriction	SE_diary, TST_diary, SOL_diary, PSQI, Rem, Res, Comp	Guided iCBT-I	SHE, SR, SK, Cog_restruct, R_PMR	6		Psychologist / Students / Therapists	59 - 49	48.7 (13.8)	59.3	11.1 (9.6)
							WL					59 - 53	50.1 (11.9)	81.4	12.6 (10.7)
Vincent & Lewycky 2009	RDC	118 - 69	Excluded	Partially excluded	No restriction	ISI, TST_diary, SOL_diary, WASO_diary, SE_diary, SQ_diary, Comp	Unguided iCBT-I	SHE, SR, Cog_restruct, Cog_problem, R_PMR, R_image	5		None	59 - 40	NA (NA)	67.8	NA
							WL					59 - 39	NA (NA)	66.1	NA
Wong et al. 2021	DSM-5	210 - 159	Partially excluded	Partially excluded	No restriction	ISI, Rem, Comp	Group	SHE, SR, SK, Cog_restruct, R	4	240 minutes total	Nurse	70 - 49	38.2 (15.8)	65.7	NA
							Unguided iCBT-I	SHE, SR, SK, Cog_restruct, R				70 - 56	36.9 (14.7)	65.9	NA
							SHE	SHE				70 - 54	39.6 (16.3)	78.6	NA

Note. DSM= Diagnostic and Statistical Manual of Mental Disorders ; ICSD = International Classification of Sleep Disorders ; RDC = Research Diagnostic Criteria for an Insomnia Disorder
ISI = Insomnia Severity Index; ISQ= Insomnia Symptom Questionaire ; SCI = Sleep Condition Indication; SII = Sleep Impairment Index; PSQI = Pittsburg Sleep Quality Index;
TST_diary = total sleep time measured via sleep diary; SE_diary = sleep efficiency measured via sleep diary; SOL_diary = sleep onset latency measured via sleep diary; WASO_diary = measured via sleep diary
TST_act = total sleep time measured via actigraphy; SE_act = sleep efficiency measured via actigraphy; SOL_act = sleep onset latency measured via actigraphy; WASO_act = measured via actigraphy
TST_psg = total sleep time measured via polysomnography; SE_psg = sleep efficiency measured via polysomnography; SOL_psg = sleep onset latency measured via polysomnography; WASO_psg = measured via polysomnography
TST_PSQI = total sleep time measured via the Pittsburg Sleep Quality Index; SE_PSQI = sleep efficiency measured via the Pittsburg Sleep Quality Index; SOL_PSQI = sleep onset latency measured via the Pittsburg Sleep Quality Index; WASO_PSQI = measured via the Pittsburg Sleep Quality Index
Rem = Remission; Res = Response; Comp = Intervention completion
F2F = Individual Onsite CBT-I; group = group delivered CBT-I; iCBT-I = internet-delivered CBT-I; smartphone = smartphone-delivered CBT-I; SHE = sleep hygiene education: ActCon = active contact control; TAU = treatment as Usual, WL= Waitlist
SHE= sleep hygiene education/psychoeducation; SR = sleep restriction ; SK = stimulus control; Cog = Cognitive therapy; Cog_restruc = cognitive restructuring; cog_problem = systematic problem solving; Cog_control = cognitive control (worry chair), Cog_PI = paradoxical intention; R = Relaxation; R_PMR = progressive muscle relaxation; R_auto = autogenic training; R_mindful = mindfulness; R_image = imagery training * = if different numbers for different outcome measures are indicated in the study, here the highest is shown

Supplementary Figure S1. Network plots and forest plots secondary outcomes

Subjective sleep quality

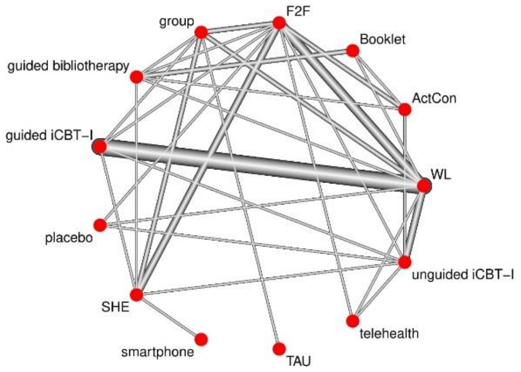

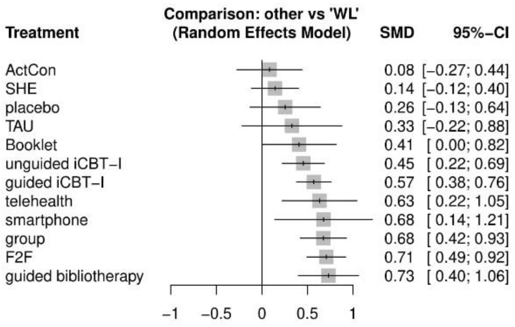

Subjective total sleep time

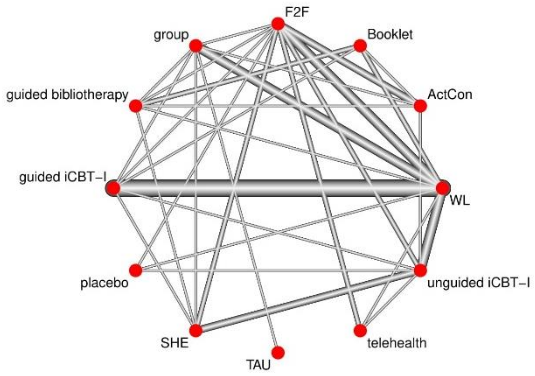

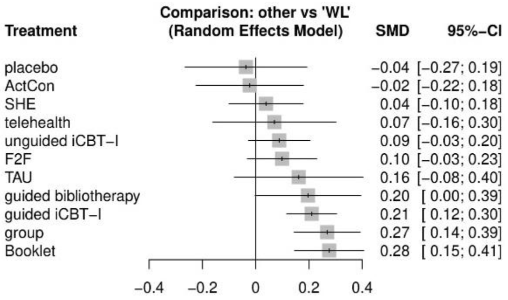

Subjective sleep efficiency

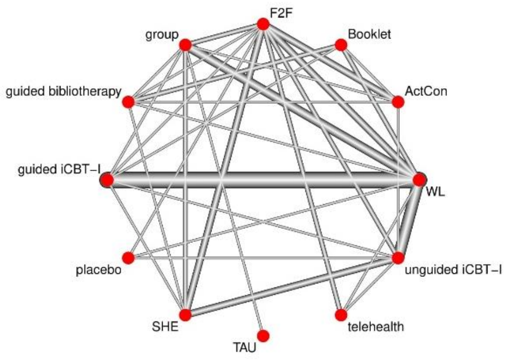

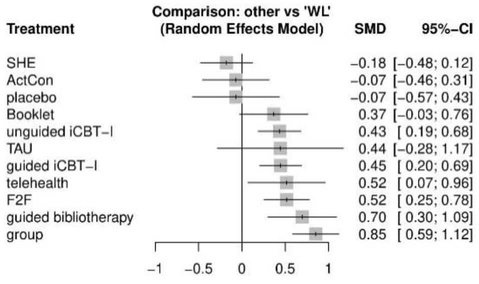

Subjective sleep onset latency

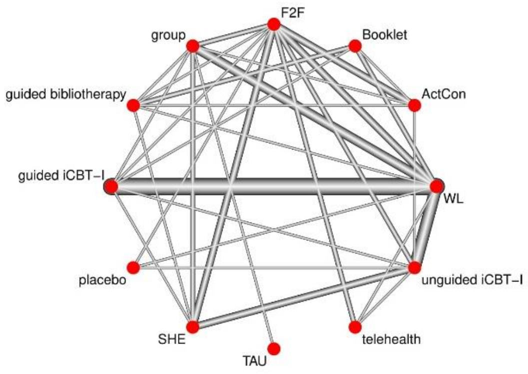

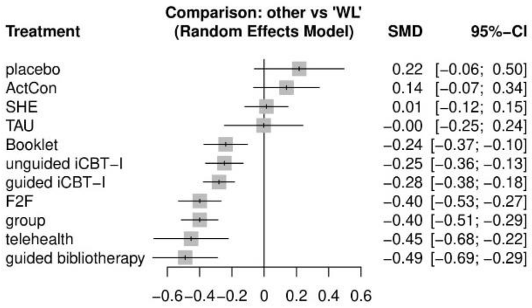

Subjective wake after sleep onset

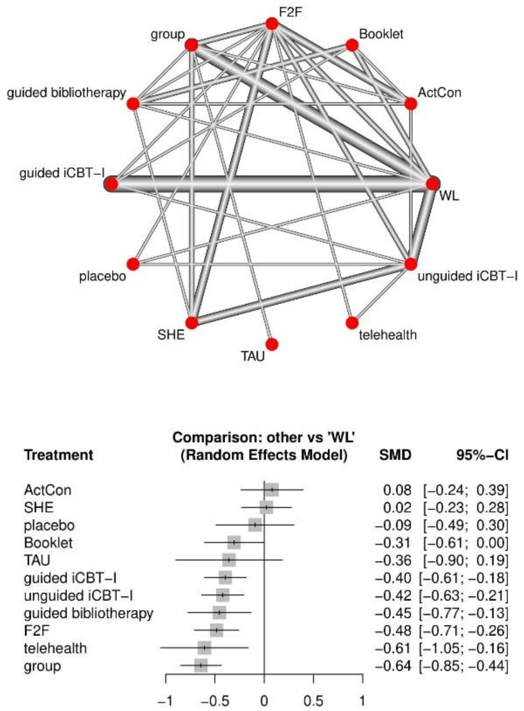

Objective total sleep time

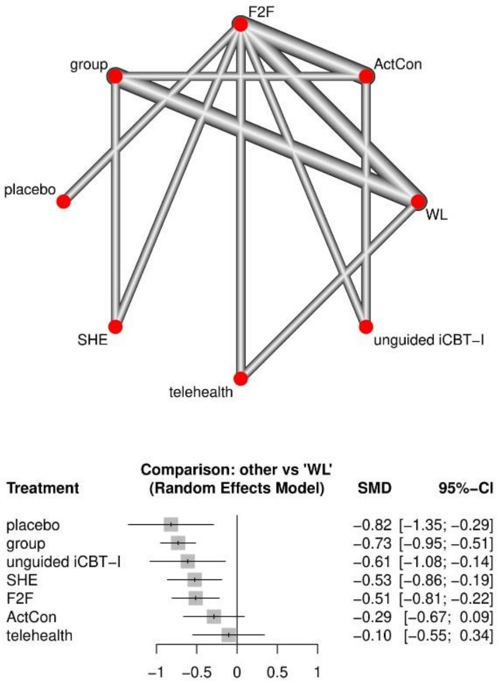

Objective sleep efficiency

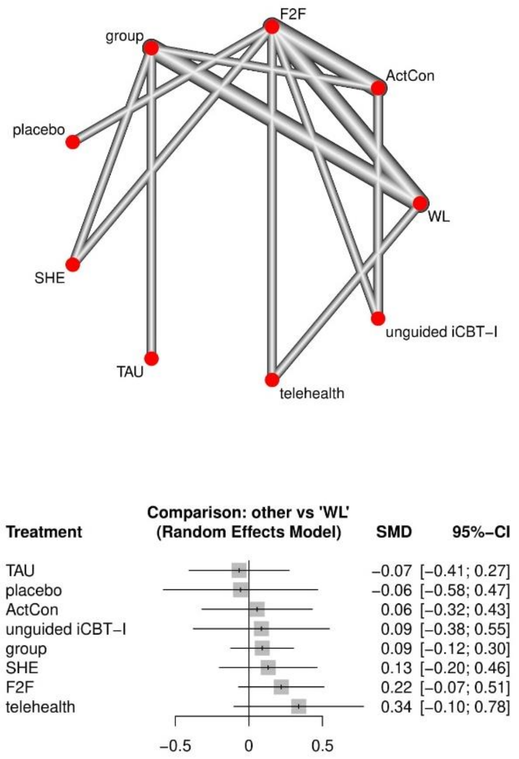

Objective sleep onset latency

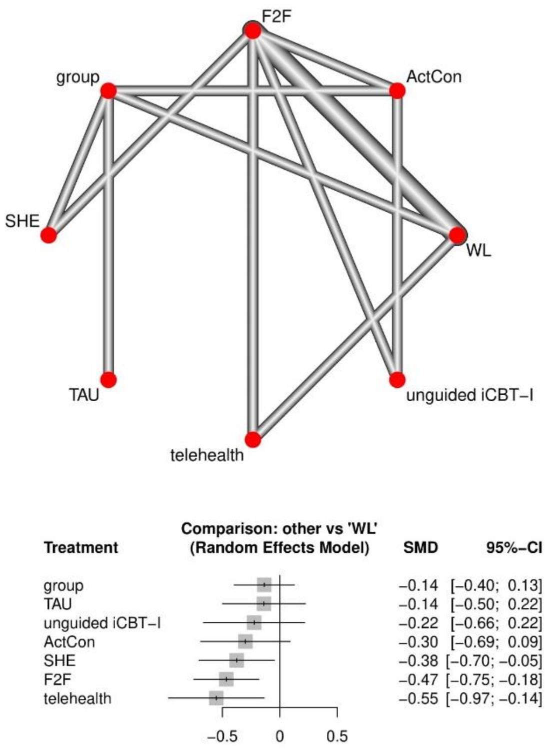

Objective wake after sleep onset

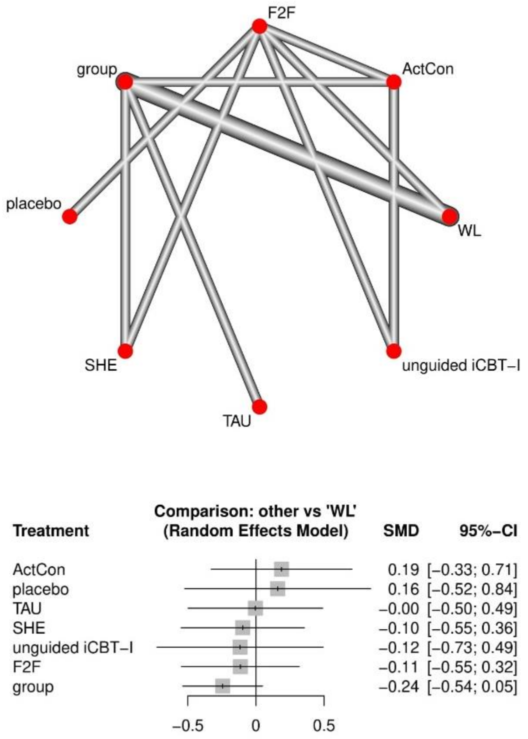

Response rates

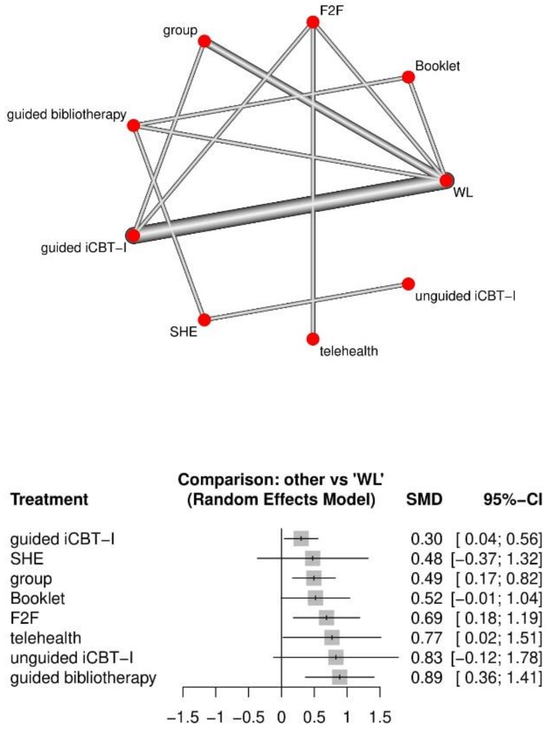

Remission rates

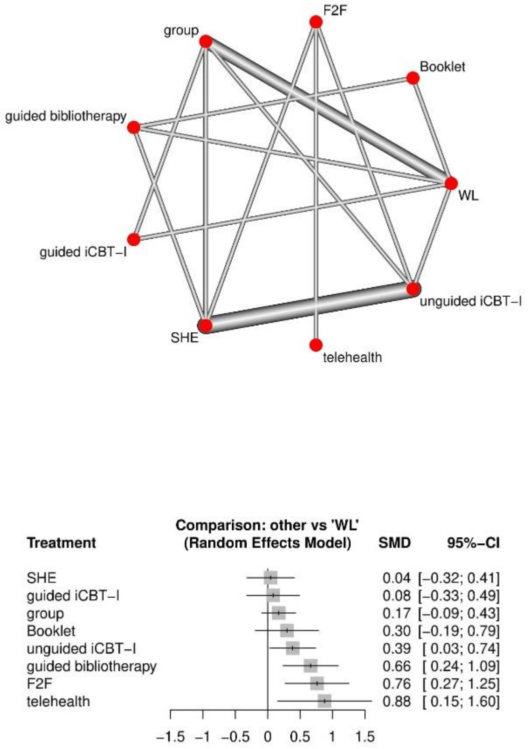

Intervention completion rates

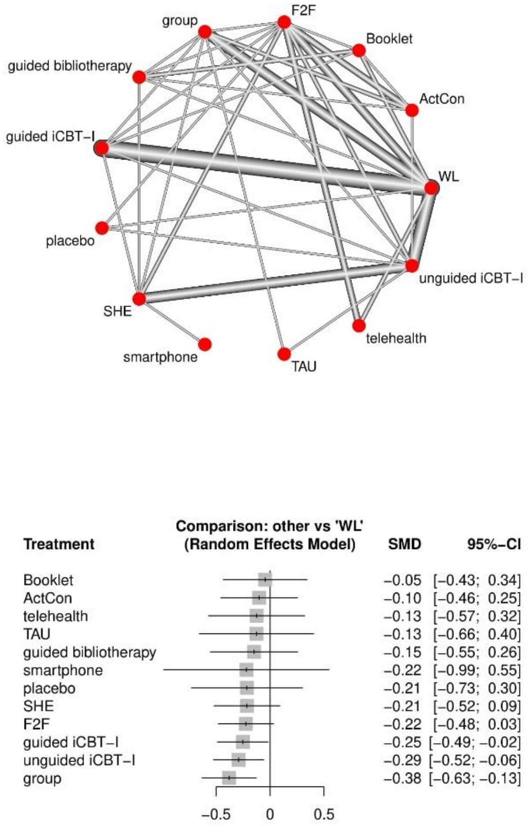

Supplementary Figure S2. Net heat plots

In the net heat plots, the network estimations of the pairwise comparisons are displayed in the rows, and the contribution of the respective design to the estimation is displayed in the columns. The area of the grey squares is proportional to the contribution of the respective design to the estimation. On the diagonal, red colors indicate that the respective design is responsible for between-design heterogeneity, whereas outside of the diagonal red colors indicates inconsistencies of the estimations of the respective design with other estimates (Schwarzer et al., 2015b). Pairwise comparisons corresponding to three-arm designs are designated by "_".

Abbreviations: ActCon = active contact control; Booklet = unguided bibliotherapy; F2F = Individual Onsite CBT-I; group = group delivered CBT-I; guided bib = guided bibliotherapy; guided iCB = guided internet-delivered CBT-I; SHE = sleep hygiene education; smartphone = smartphone-delivered CBT-I; TAU = treatment as Usual; unguided i= unguided internet-delivered CBT-I;WL= waiting list

Insomnia severity

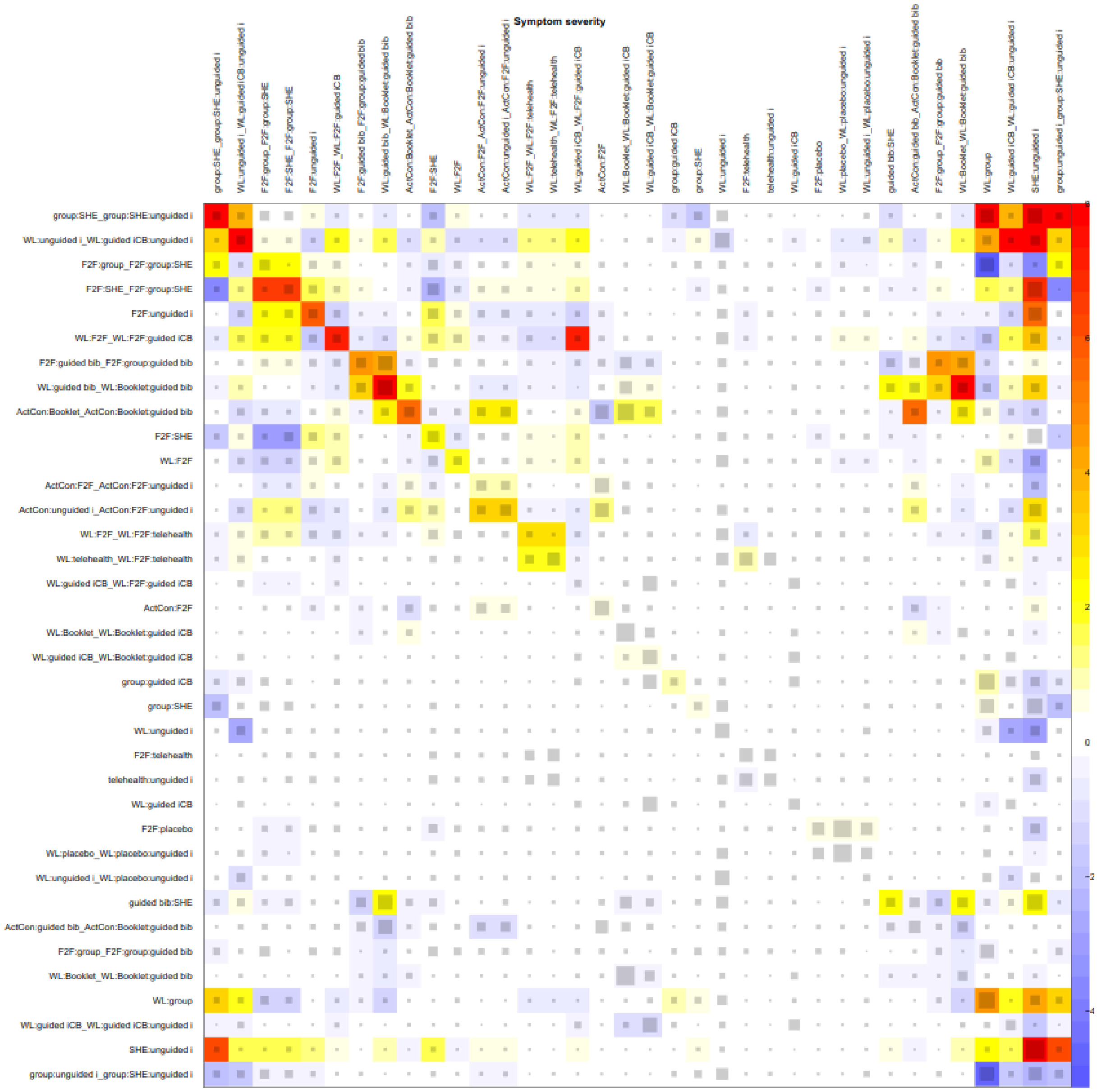

Exemplary interpretation of the net heat plot for insomnia severity:

There are several large grey squares outside of the diagonal indicating the importance of indirect evidence in the estimation of the network estimates. For example, for the estimation of unguided iCBT-I compared to WL (design from a three-armed study comparing unguided iCBT-I, WL, and placebo), the estimation of unguided iCBT-I compared to WL is an important source of indirect evidence. The following treatment comparisons contribute the most to the between-study heterogeneity: unguided iCBT-I compared to WL (design from a three-armed study comparing unguided iCBT-I, WL, and placebo), unguided iCBT-I compared to WL, group-delivered CBT-I compared to SHE (design from a three-armed study comparing group-delivered CBT-I, unguided iCBT-I, and SHE), and unguided iCBT-I compared to WL (design from a three-armed study comparing unguided iCBT-I, guided iCBT-I, and WL). Moreover, inconsistencies between indirect and direct evidence were observed for these estimates.

Subjective sleep quality

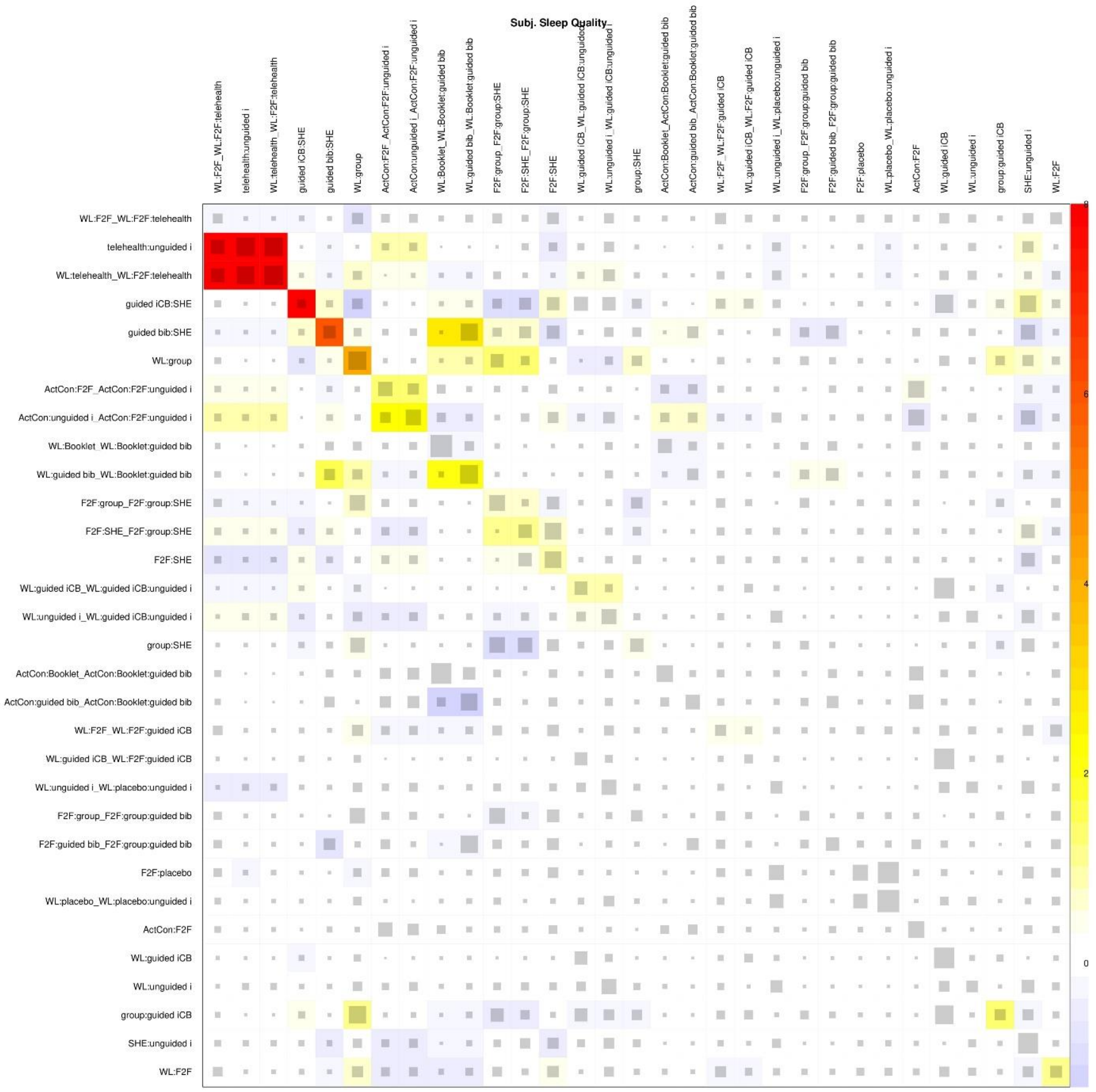

Subjective total sleep time

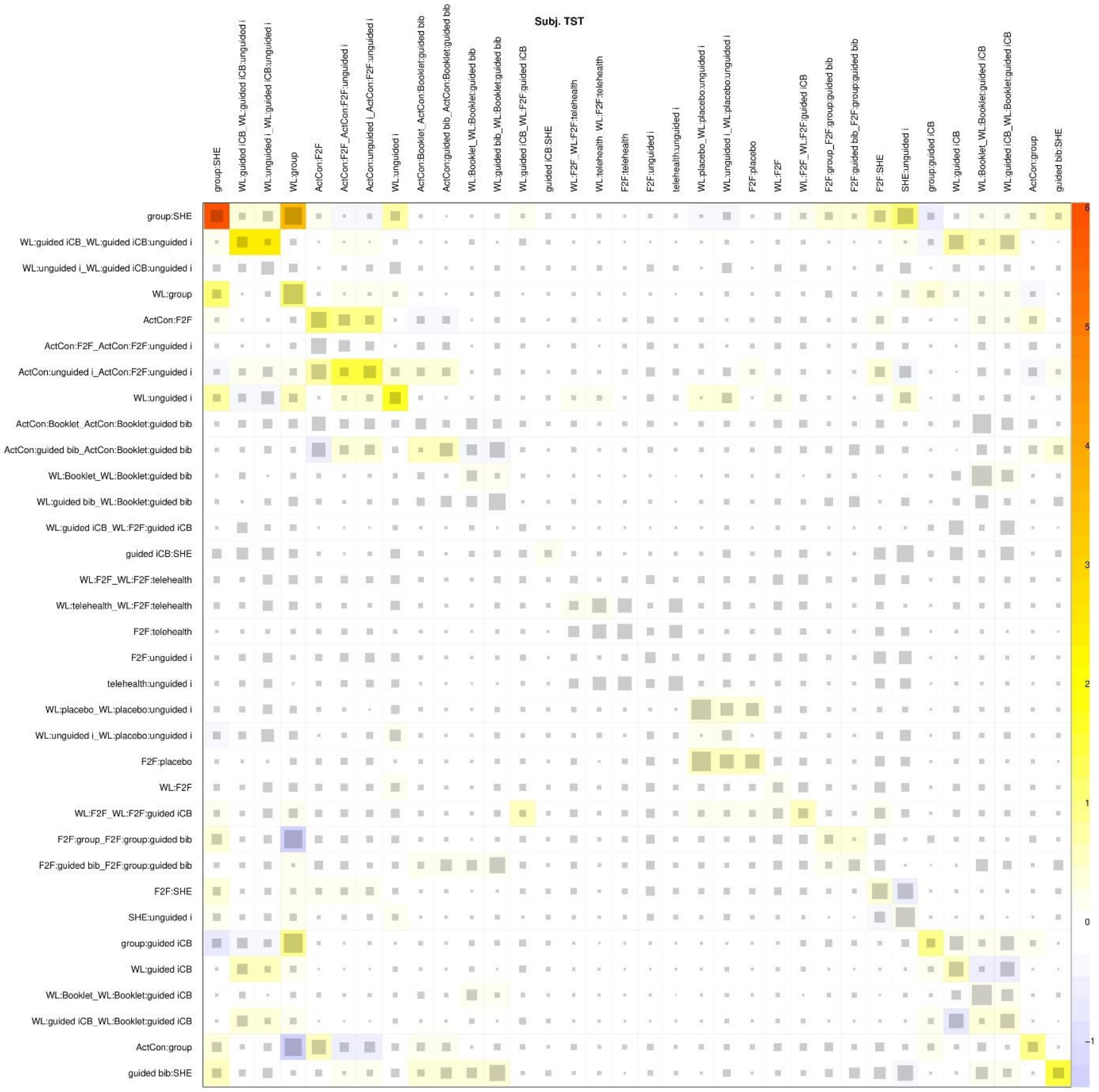

Subjective sleep efficiency

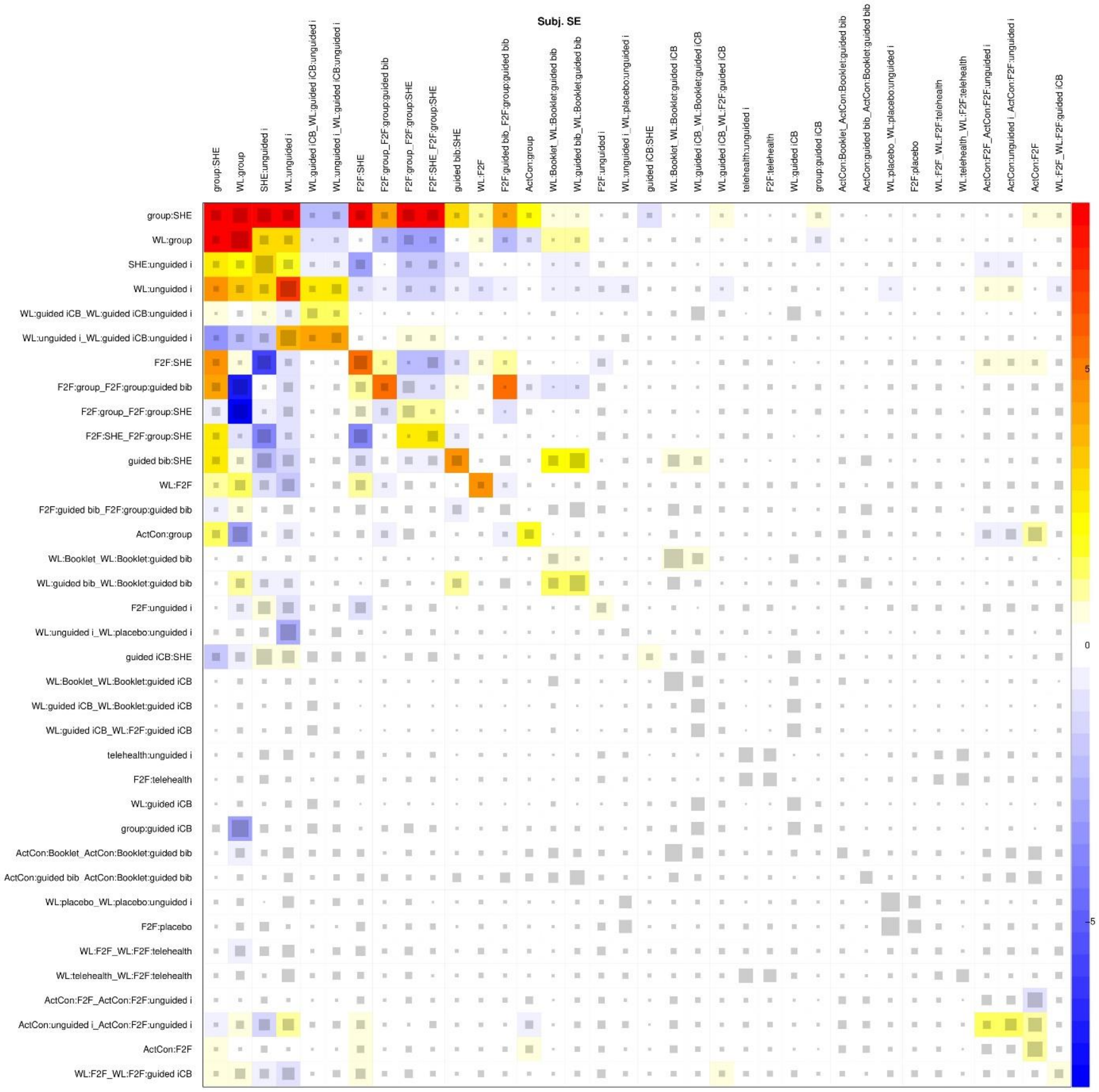

Subjective sleep onset latency

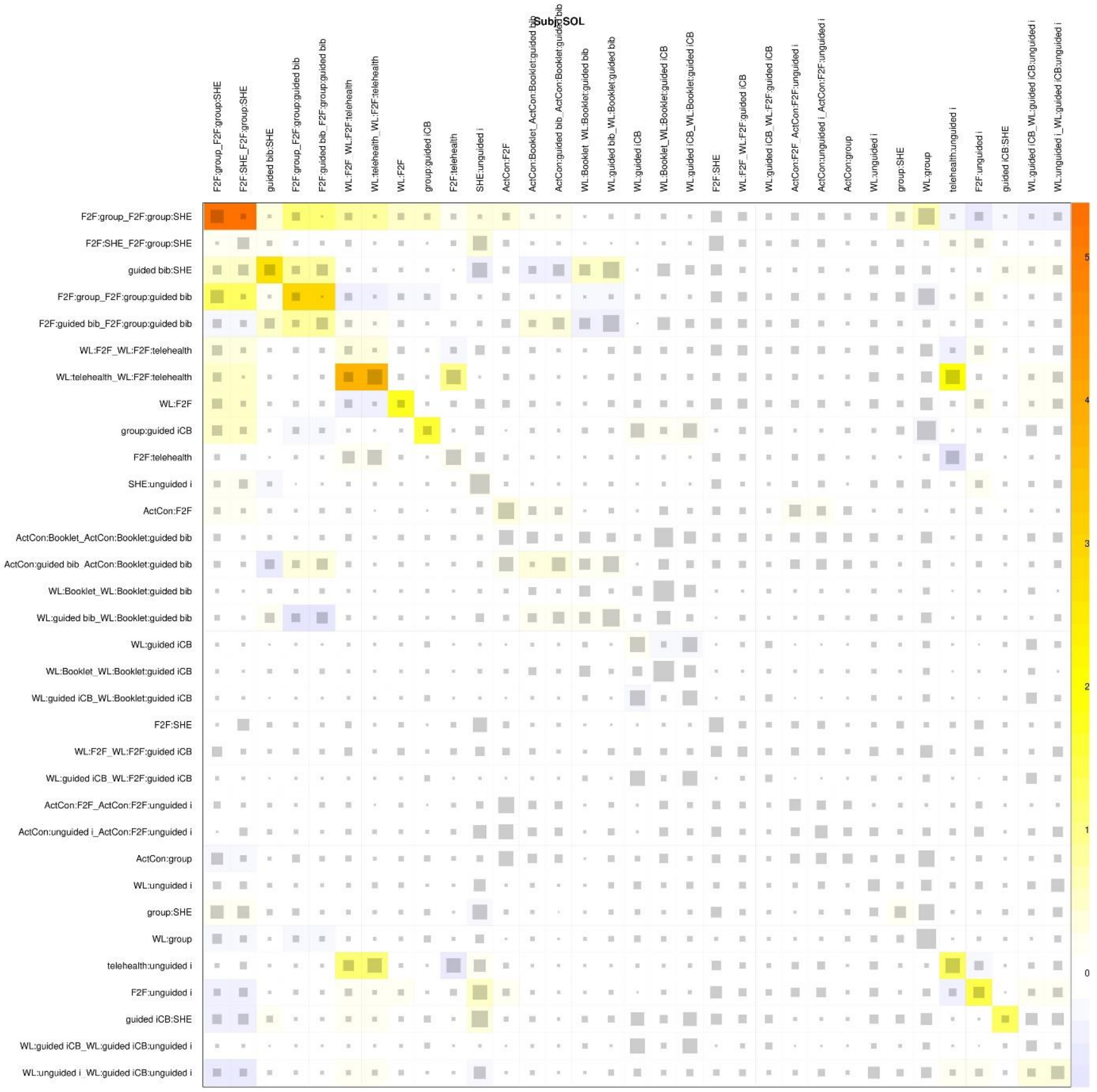

Subjective wake after sleep onset

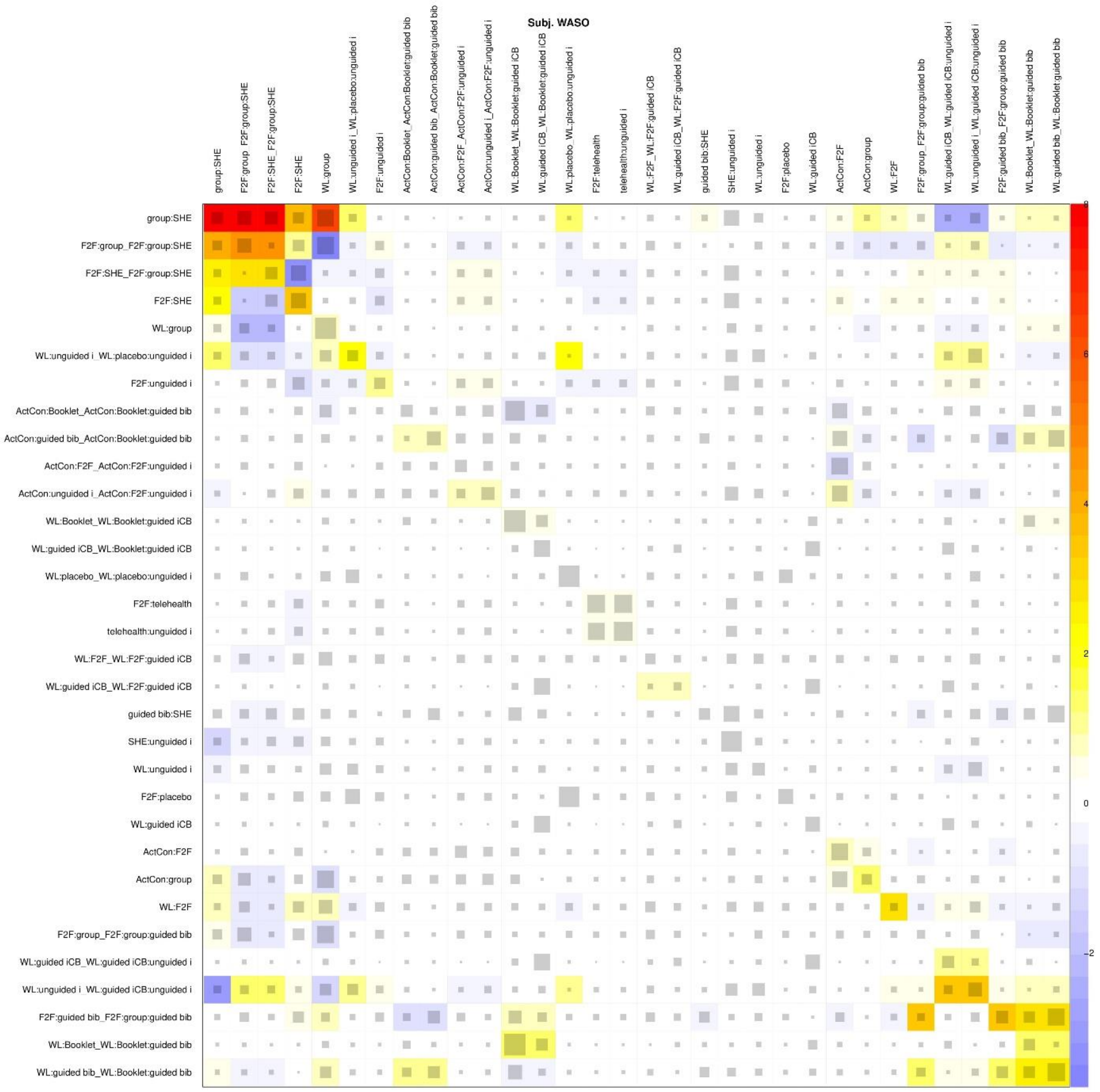

Objective total sleep time

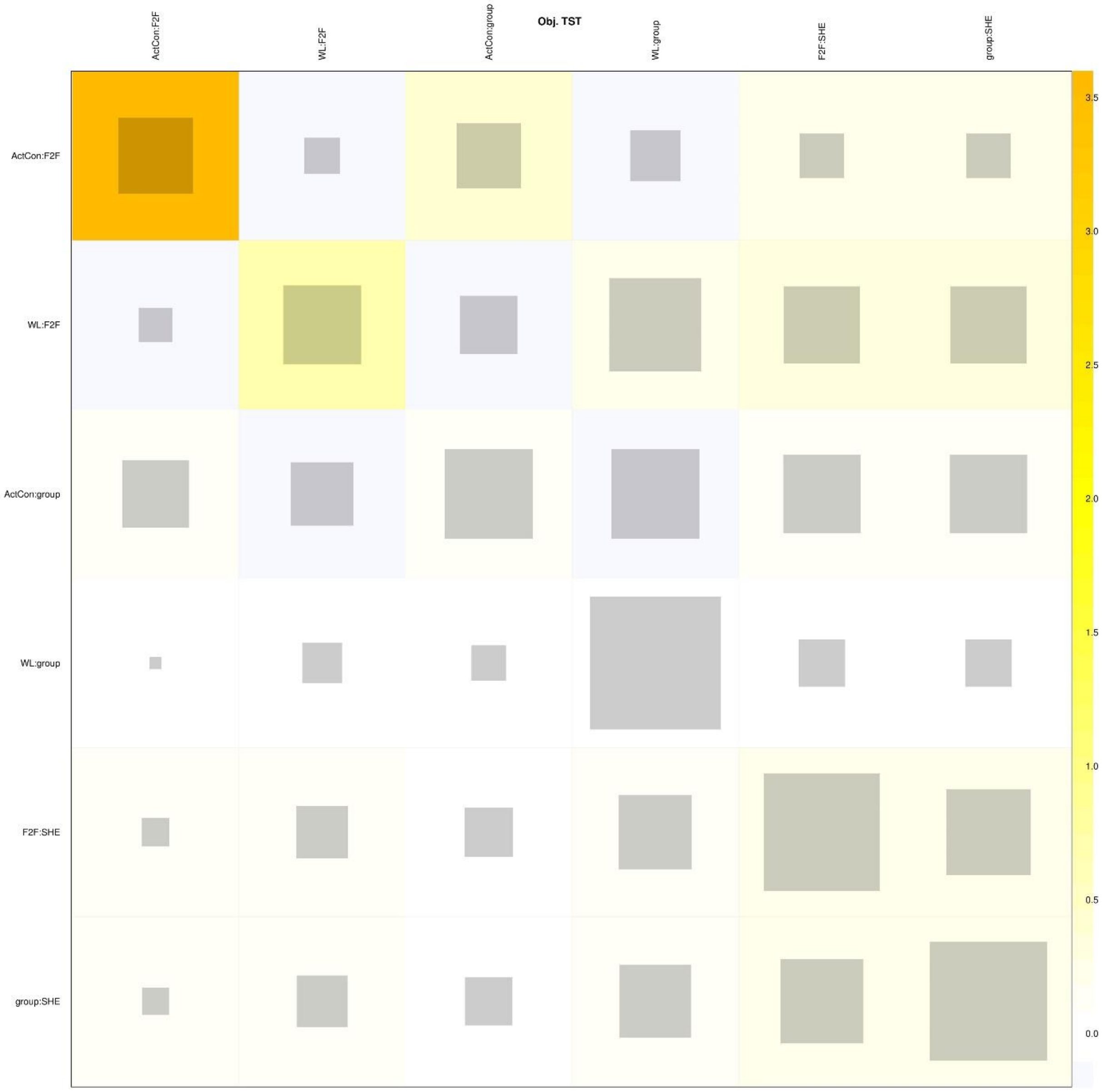

Objective sleep efficiency

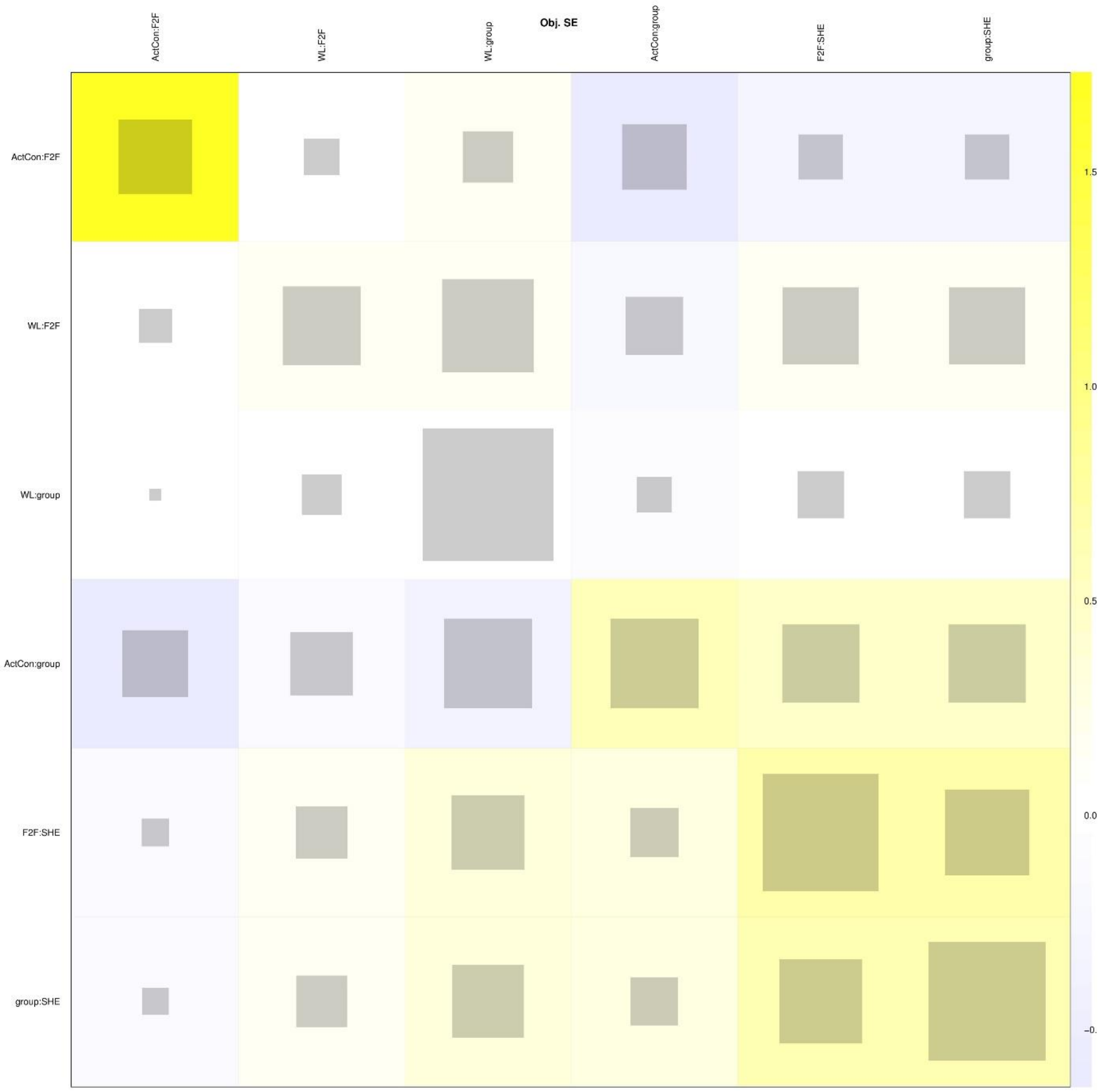

Objective sleep onset latency

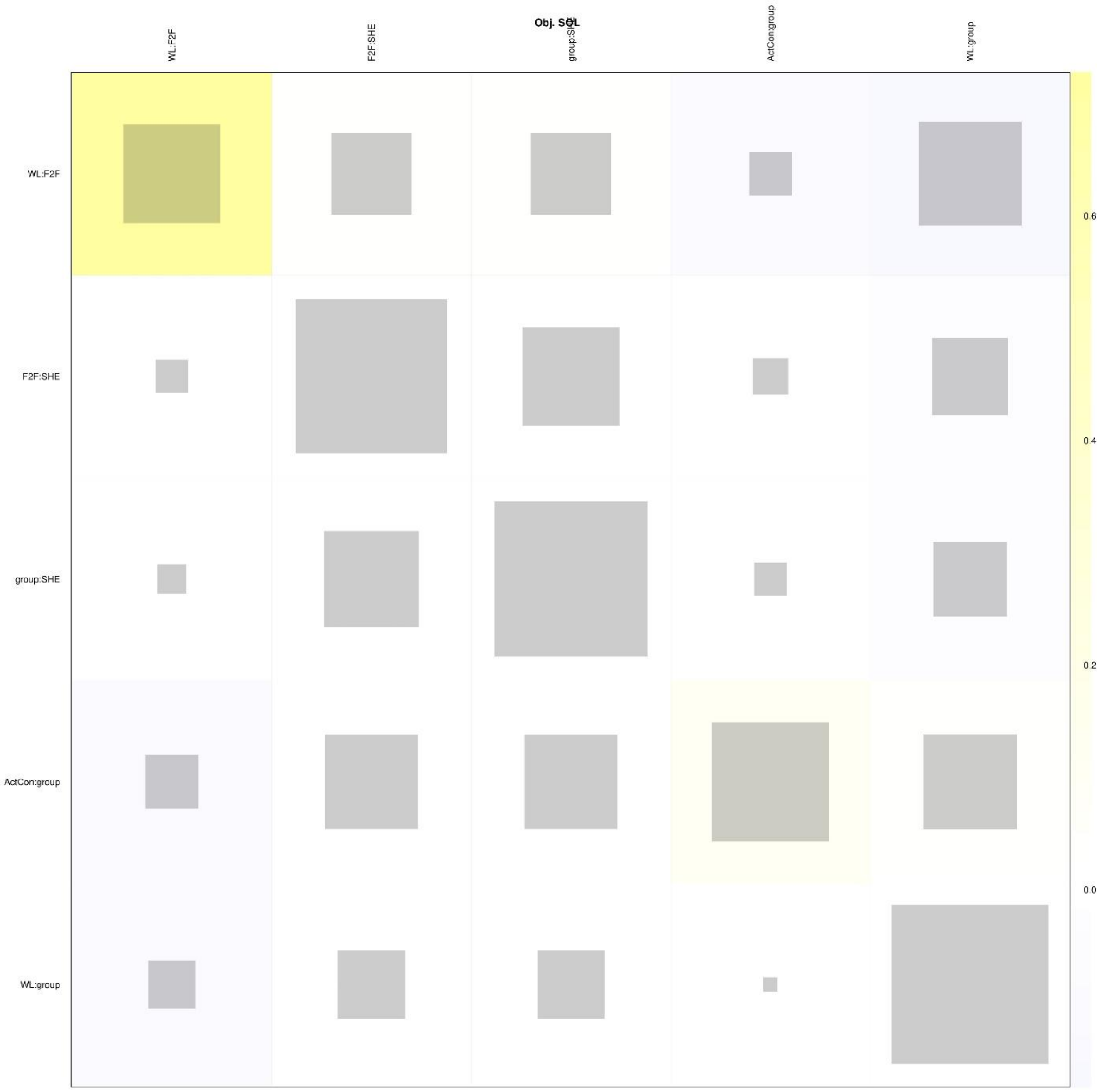

Objective wake after sleep onset

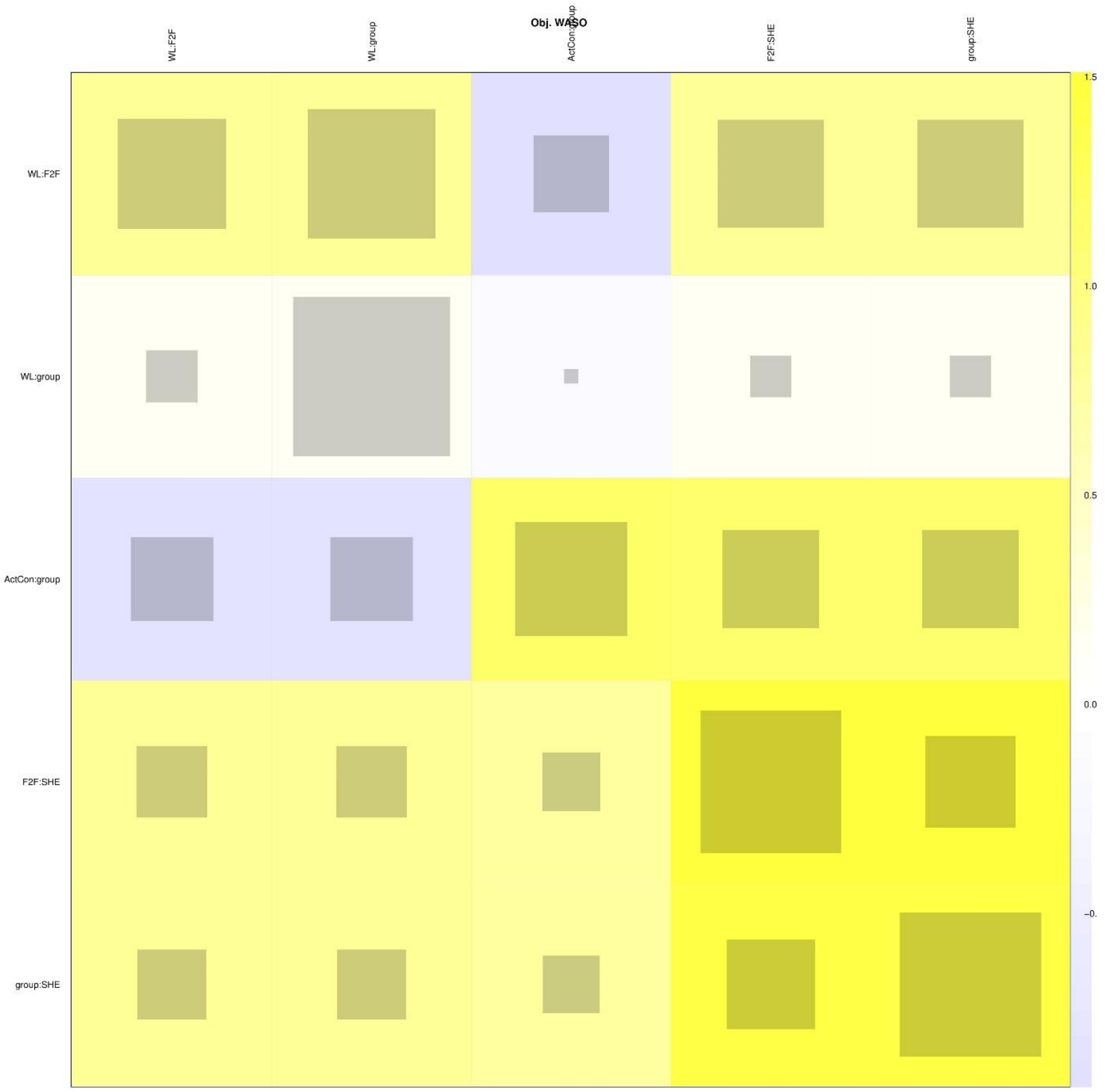

Response rates

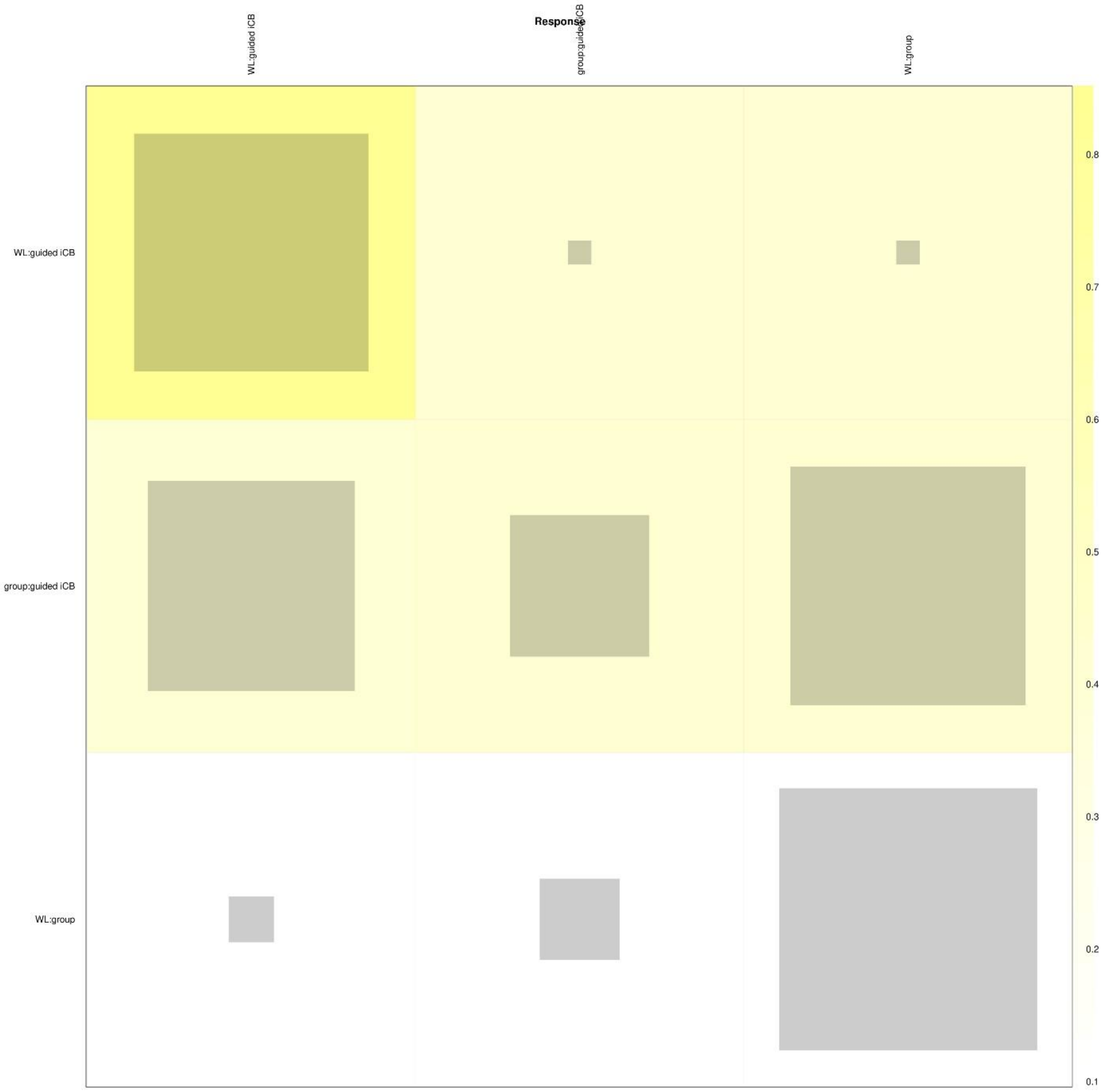

Remission rates

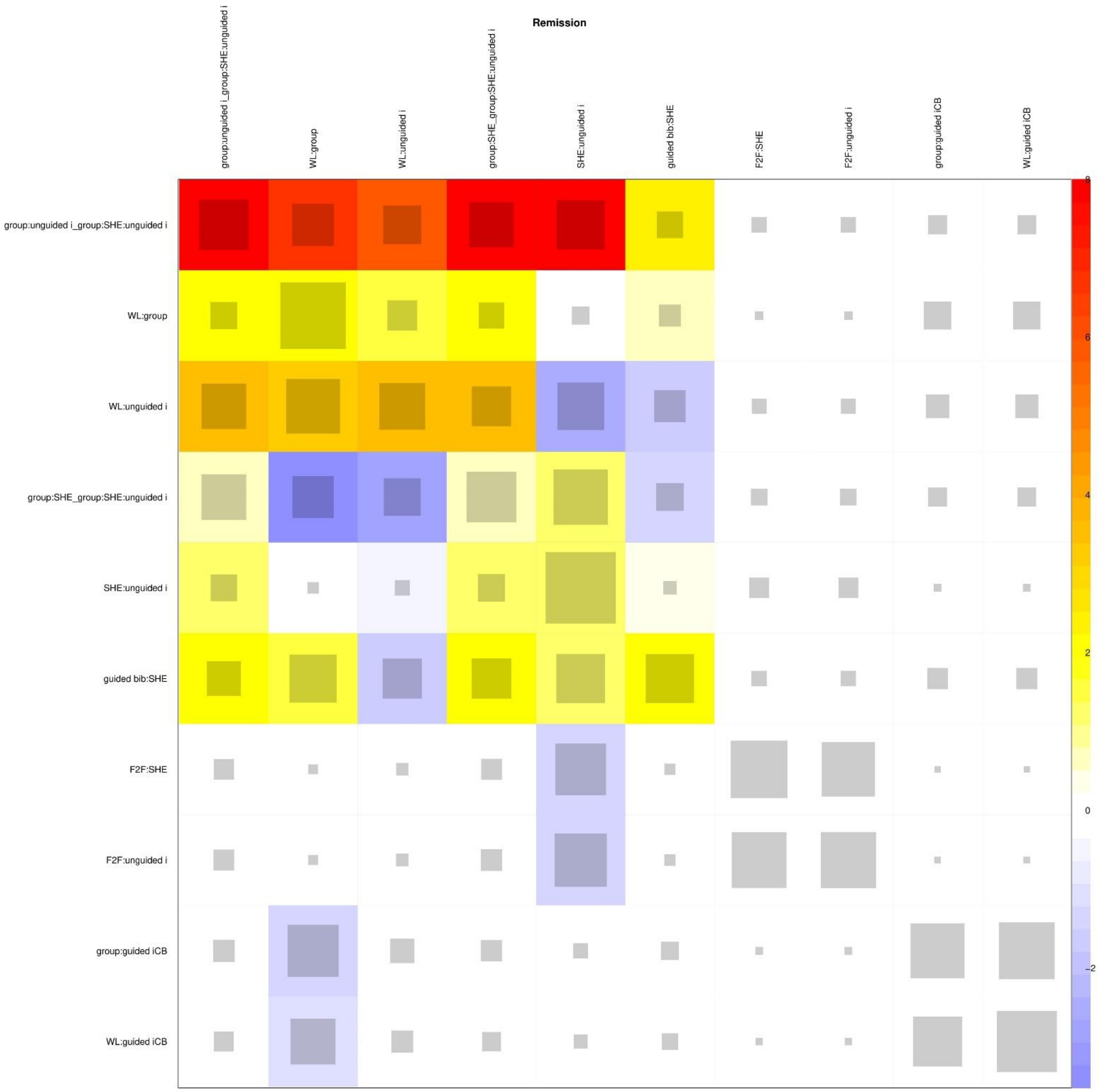

Intervention completion rates

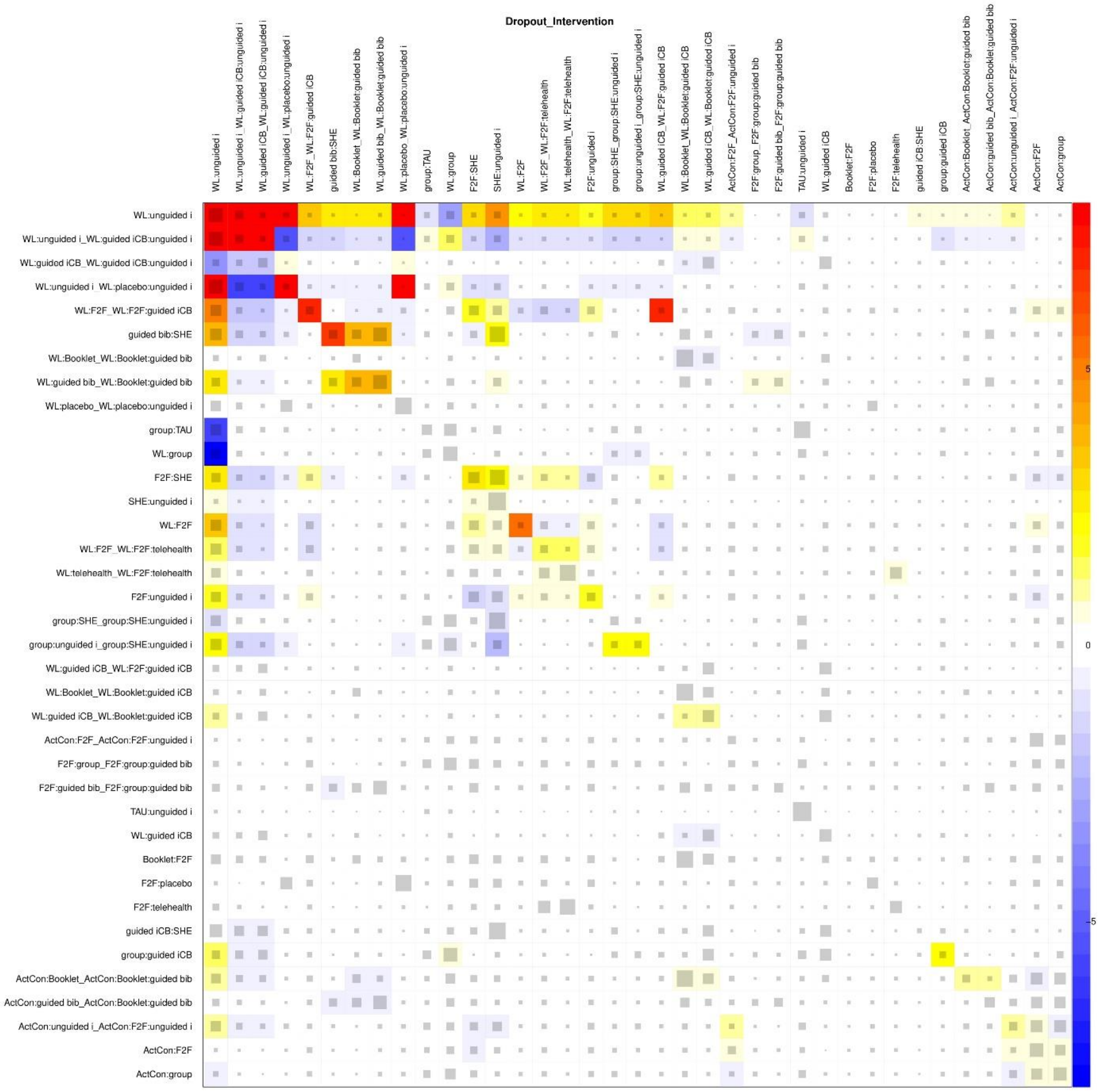

Supplementary Figure S3. Risk of Bias- summary figure

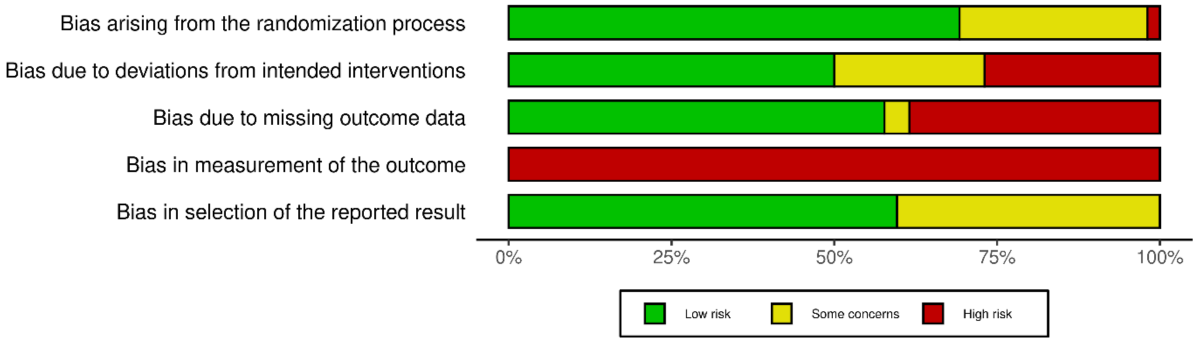

Supplementary Figure S4. Risk of Bias- detailed figure

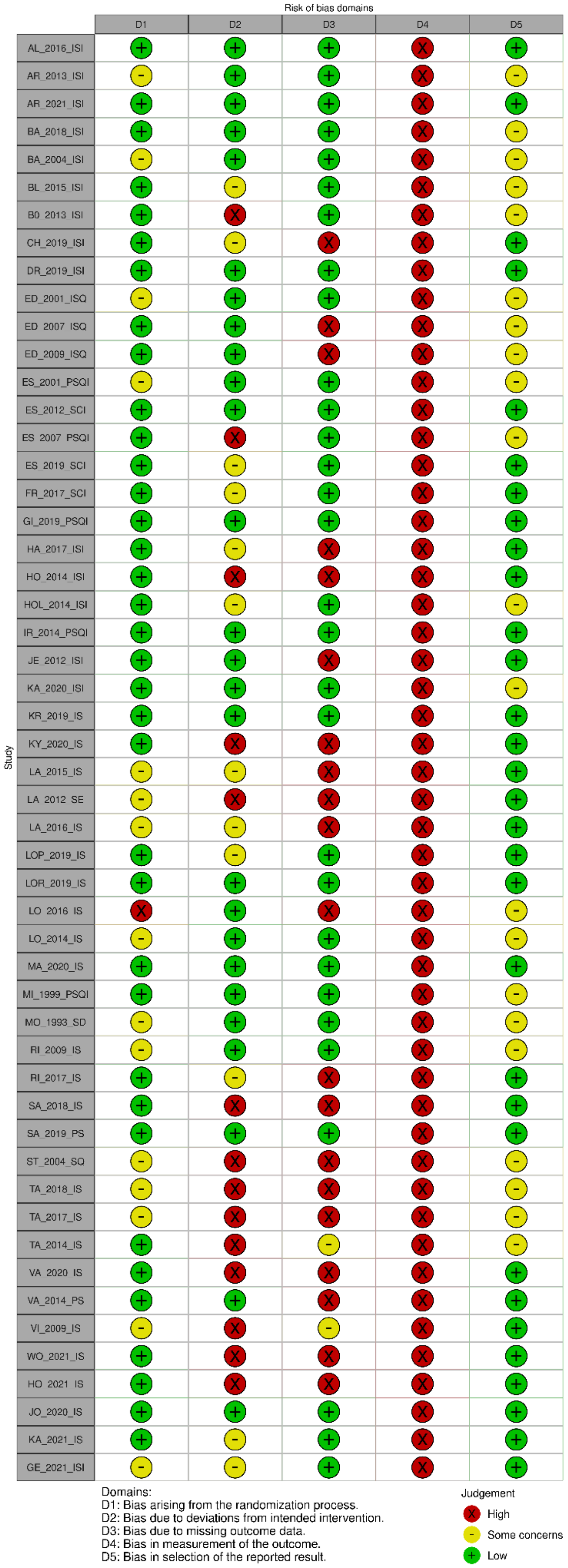

Supplementary Figures S5. Comparison-adjusted funnel plots

Insomnia severity

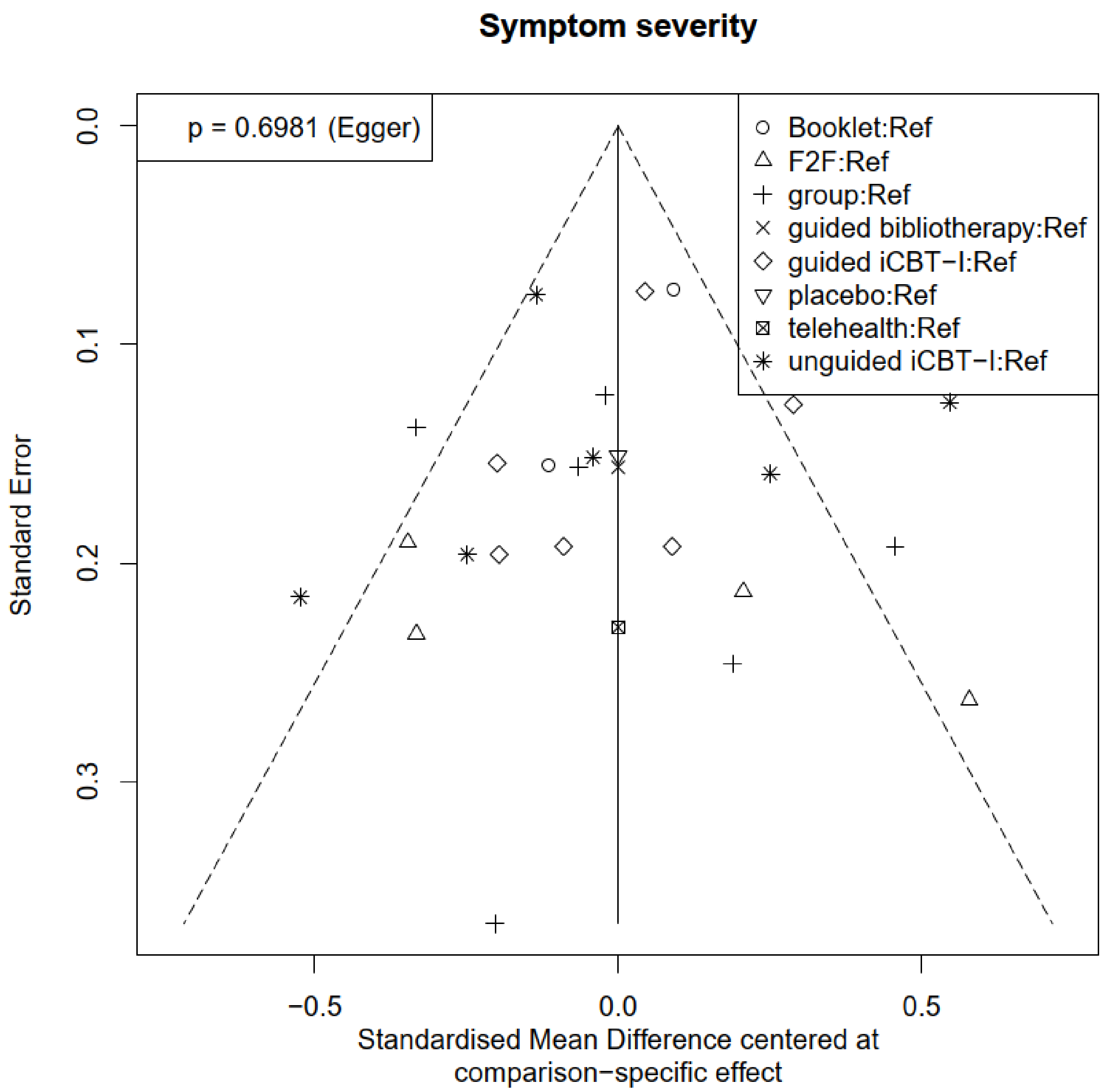

Subjective sleep quality

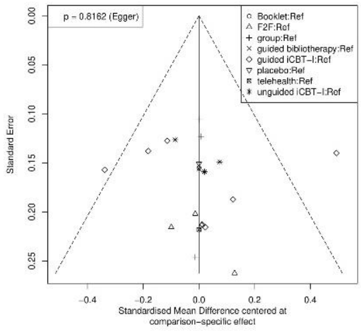

Subjective total sleep time

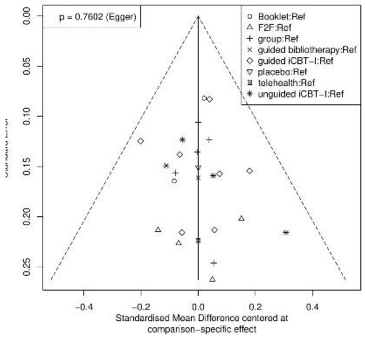

Subjective sleep efficiency

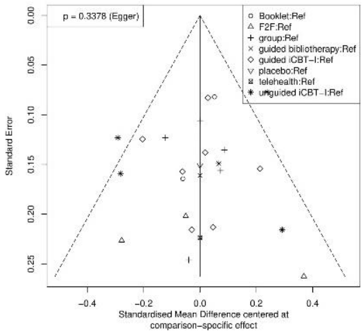

Subjective sleep onset latency

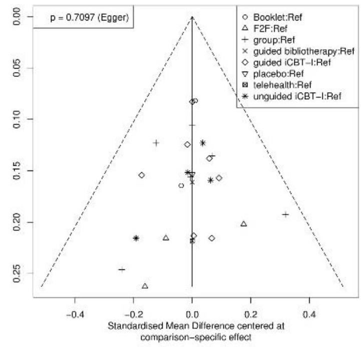

Subjective wake after sleep onset

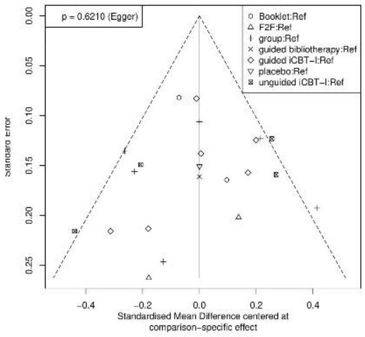

Objective total sleep time

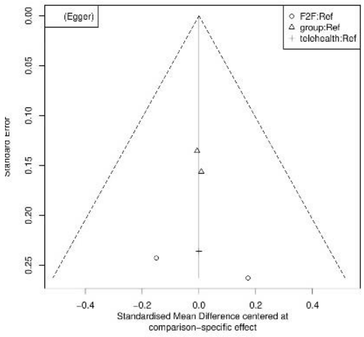

Objective sleep efficiency

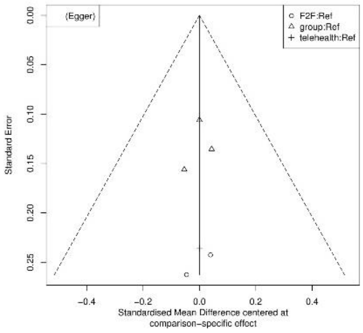

Objective sleep onset latency

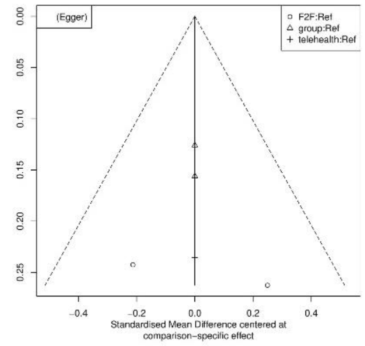

Objective wake after sleep onset

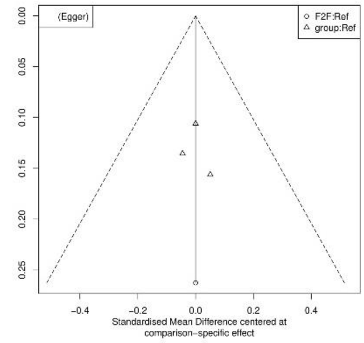

Supplement: Supplementary file 1 — Supplementary Information. [file 41598_2023_28853_MOESM1_ESM.pdf]
